# Enhanced anti-inflammatory effects of mesenchymal stromal cells mediated by the transient ectopic expression of CXCR4 and IL10

**DOI:** 10.1186/s13287-021-02193-0

**Published:** 2021-02-12

**Authors:** Rosario Hervás-Salcedo, María Fernández-García, Miriam Hernando-Rodríguez, Oscar Quintana-Bustamante, Jose-Carlos Segovia, Marcio Alvarez-Silva, Mariano García-Arranz, Pablo Minguez, Victoria del Pozo, Marta Rodríguez de Alba, Damián García-Olmo, Carmen Ayuso, María Luisa Lamana, Juan A. Bueren, Rosa María Yañez

**Affiliations:** 1 Hematopoietic Innovative Therapies Division, Centro de Investigaciones Energéticas, Medioambientales y Tecnológicas (CIEMAT), Av. Complutense 40, 28040 Madrid, Spain; 2 Centro de Investigación Biomédica en Red de Enfermedades Raras (CIBERER), Madrid, Spain; 3 Instituto de Investigaciones Sanitarias Fundación Jiménez Díaz (IIS-FJD), Madrid, Spain; 4 Stem Cell and Bioengineering Laboratory, Universidade Federal de Santa Catarina, Florianópolis, Brazil; 5 Centro de Investigación Biomédica en Red de Enfermedades Respiratorias (CIBERES), Madrid, Spain

**Keywords:** Mesenchymal stromal cells, CXCR4, IL10, mRNA-modified MSCs, Inflammation, MSC homing

## Abstract

**Background:**

Mesenchymal stromal cells (MSCs) constitute one of the cell types most frequently used in cell therapy. Although several studies have shown the efficacy of these cells to modulate inflammation in different animal models, the results obtained in human clinical trials have been more modest. Here, we aimed at improving the therapeutic properties of MSCs by inducing a transient expression of two molecules that could enhance two different properties of these cells. With the purpose of improving MSC migration towards inflamed sites, we induced a transient expression of the C-X-C chemokine receptor type 4 (CXCR4). Additionally, to augment the anti-inflammatory properties of MSCs, a transient expression of the anti-inflammatory cytokine, interleukin 10 (IL10), was also induced.

**Methods:**

Human adipose tissue-derived MSCs were transfected with messenger RNAs carrying the codon-optimized versions of CXCR4 and/or IL10. mRNA-transfected MSCs were then studied, first to evaluate whether the characteristic phenotype of MSCs was modified. Additionally, in vitro and also in vivo studies in an LPS-induced inflamed pad model were conducted to evaluate the impact associated to the transient expression of CXCR4 and/or IL10 in MSCs.

**Results:**

Transfection of MSCs with CXCR4 and/or IL10 mRNAs induced a transient expression of these molecules without modifying the characteristic phenotype of MSCs. In vitro studies then revealed that the ectopic expression of CXCR4 significantly enhanced the migration of MSCs towards SDF-1, while an increased immunosuppression was associated with the ectopic expression of IL10. Finally, in vivo experiments showed that the co-expression of CXCR4 and IL10 increased the homing of MSCs into inflamed pads and induced an enhanced anti-inflammatory effect, compared to wild-type MSCs.

**Conclusions:**

Our results demonstrate that the transient co-expression of CXCR4 and IL10 enhances the therapeutic potential of MSCs in a local inflammation mouse model, suggesting that these mRNA-modified cells may constitute a new step in the development of more efficient cell therapies for the treatment of inflammatory diseases.

**Supplementary Information:**

The online version contains supplementary material available at 10.1186/s13287-021-02193-0.

## Background

Mesenchymal stromal cells (MSCs) are multipotent adult stromal cells with immunomodulatory effects on activated lymphoid cells [1–3], including T cells [4], B cells [5], natural killer cells [6], and dendritic cells [7]. MSCs also display the ability to home on inflamed sites, where they can modulate inflammatory reactions [8] and contribute to the repair of injured tissues [9].

In animal models, MSCs have shown their efficacy both in regenerative medicine and also in inflammatory and autoimmune disease models [10–14]. In phase I/II clinical trials, MSCs have demonstrated a safety profile and preliminary evidence of clinical benefit in different diseases such as steroid-resistant graft versus host disease (GVHD) [15], severe systemic lupus erythematosus [16], complex perianal fistulas [17], knee osteoarthritis [18], or chronic complete paraplegia [19]. Despite the results obtained in animal models and early-phase clinical trials, only in three phase III clinical trials the therapeutic efficacy of MSCs has shown statistical significance over standard therapies. These include the treatment of complex perianal fistulas (NCT00475410), steroid-refractory GVHD in children, and chronic advanced ischemic heart failure (NCT01768702) [20–22]. indicating the convenience of enhancing the therapeutic efficacy of these cells.

To enhance the therapeutic potential of MSCs, previous studies have proposed their modification either with drugs or by means of the transient or stable modification of these cells with RNA or DNA sequences capable of promoting the expression of homing or therapeutic molecules [23–25].

In this respect, the C-X-C chemokine receptor type 4 (CXCR4), the receptor of the stromal cell-derived factor-1 (SDF-1), plays an important role in cell migration to inflamed sites [26, 27]. Because only a low proportion of MSCs expresses CXCR4 in their membrane [28, 29] and given that this expression is further reduced during the ex vivo expansion of these cells [28, 30], homing receptors such as CXCR4 have been ectopically expressed in MSCs to enhance their migration towards inflamed tissues in different animal models [31, 32]. Other studies have described that the transfer of genes encoding for anti-inflammatory cytokines improves the potential of MSCs to modulate inflammation diseases [32–34]. In particular, IL10, a strong anti-inflammatory cytokine, has been ectopically expressed in MSCs to improve the immunomodulatory properties of these cells [23, 35, 36]. Since CXCR4 and IL10 exert independent and potentially synergistic effects on MSCs, here, we aimed at investigating the beneficial effects associated to the ectopic expression of these molecules, either individually or combined.

Because the therapeutic efficacy of MSCs appears to be mediated through early events occurring during the first days/hours following their systemic infusion [9], strategies based on the transfection of messenger RNA (mRNA) constitute attractive approaches for the engineering of these cells due to its simplicity, transient, and rapid protein translation that take place after mRNA transfection [36–39]. Additionally, mRNA transfection is highly efficient and non-toxic in MSCs, and it is compatible with the ectopic co-expression of several mRNAs. This technology could thus be appropriate both for improving MSC homing to inflamed tissues and also for inducing a transient delivery of anti-inflammatory cytokines.

In order to improve the therapeutic efficacy of MSCs in inflammatory diseases in this study, we constructed independent mRNAs carrying codon-optimized versions of CXCR4 and IL10. Additionally, a bicistronic mRNA carrying both the CXCR4 and IL10 coding sequences was generated. Our results demonstrate the enhanced in vitro and in vivo efficacy of mRNA-modified Ad-MSCs to migrate and exert immunosuppressive and anti-inflammatory responses as compared to unmodified MSCs. Our preclinical studies strongly suggest that mRNA-transfected MSCs, particularly those co-expressing IL10 and CXCR4, may improve the clinical efficacy of MSC-based cell therapies as compared to standard unmodified MSCs.

## Materials and methods

### mRNA synthesis

Codon-optimized sequences of human *CXCR4* and *IL10* cDNAs (GeneScript, NJ, USA) were cloned into a pUC57 plasmid as monocistronic constructs or as a bicistronic construct using the E2A sequence to facilitate the co-expression of both proteins [40]. In all instances, cDNAs were driven by the T7 promoter, flanked by the 5′ and 3′ untranslated regions (UTRs) from human β-globin (HBB), and included an optimized Kozak sequence for an efficient initiation of translation [41].

The in vitro transcription of these constructs was carried out using the 5X MegaScript T7 Kit (Ambion/Invitrogen/Thermo Fisher Scientific, TX, USA). During the process, a 3′-0-Me-m7G(5′)ppp (5′)G RNA Cap Structure Analog (ARCA; New England Biolabs, MA, USA) in the 5′-end was added, and a 3′-poly(A) tail was also included using the Poly(A) Tailing Kit (Ambion/Invitrogen/Thermo Fisher Scientific, TX, USA). When a capped polyadenylated RNA was synthesized, it was purified using the columns and protocol of the RNAeasy® Plus Mini Kit (Qiagen, Hilden, Germany). Transcripts were then quantified and visualized in a 1% agarose gel.

### Generation, expansion, and characterization of Ad-MSCs

Adipose tissue samples were obtained from healthy donor lipoaspirations after informed consent in accordance with the Helsinki Declaration of 1975 (revised in 2000) and purchased from Caltag MedSystem (Buckingham, UK). The project was approved by the Ethics Committee of Hospital Fundación Jiménez Díaz (Madrid, Spain). Adipose tissue was disaggregated and digested with collagenase A (Serva, Heidelberg, Germany) at a final concentration of 2 mg/ml for 4 h at 37 °C. Digested samples were filtered through 100-μm nylon filters (BD Bioscience, NJ, USA) and centrifuged for 10 min. The cell pellet was re-suspended in Minimum Essential Medium α (ɑ-MEM; Gibco/Life Technologies/Thermo Fisher Scientific, Waltham, USA) supplemented with 5% platelet lysate (Cook Medical, IN, USA), 1% penicillin/streptomycin (Gibco/Life Technologies/Thermo Fisher Scientific, Waltham, USA), and 1 ng/ml human basic fibroblast growth factor (bFGF; Peprotech, NJ, USA). Cells were seeded at a concentration of 10,000 cells/cm^2^ in culture flasks (Corning, NY, USA) and cultured at 37 °C. For the expansion of Ad-MSCs, the cell medium was changed every 2–4 days and adherent cells were serially passaged using 0.25% trypsin/EDTA (Sigma Aldrich, St. Louis, MO, USA) upon reaching near confluence (70%–90%). For in vitro and in vivo studies, Ad-MSCs were used at passages from 4 to 8.

Ad-MSCs were immunophenotypically characterized by flow cytometry (Fortessa, BD Bioscience, NJ, USA) as described by the *Mesenchymal cell kit* (Immunostep, Salamanca, Spain). The monoclonal anti-human antibodies included in these studies were the following: CD29, CD44, CD73, CD90, CD105, CD166, CD45, CD19, HLA-DR, CD14, and CD34. Data were analyzed with FlowJo version X (FlowJo LLC, CA, USA).

The osteogenic and adipogenic differentiation ability of Ad-MSCs was determined using the NH-OsteoDiff and NH-AdipoDiff Media (Miltenyi Biotec, Bergisch Gladbach, Germany), respectively, according to the manufacturer’s protocols. Alkaline phosphatase deposits were seen after the staining with Fast BCIP/NCP (Sigma Aldrich, St. Louis, MO, USA) while lipid droplets were seen with optic microscopy (Nikon, Düsseldorf, Germany).

### mRNA transfection of Ad-MSCs

Ad-MSCs were plated and incubated for 24 h. To optimize Ad-MSC transfection, three different lipofectamines were tested according to the manufacturer’s protocols using an EGFP-mRNA: Lipofectamine™ 3000, Lipofectamine™ RNAiMax, and Lipofectamine™ Messenger (Invitrogen/Thermo Fisher Scientific, MA, USA). After optimization, each mRNA of interest was added to the Opti-MEM solution at a concentration of 1 μg/100,000 cells and mixed gently. Lipofectamine™ Messenger (5 μL/100,000 cells) was added to the same amount of the Opti-MEM medium. Both solutions were mixed and incubated for 5 min at room temperature and then incubated with the cells for different time points.

### Cell viability assay

Ad-MSCs were seeded in 96-well plates at a concentration of 4000 cells/well. Cell viability was measured using *ViaLight™ plus assay kit* (Lonza, Basel, Switzerland) in a Microplate Reader GENios (Tecan Trading AG, Männedorf, Switzerland).

### Gene expression analysis

RNA from Ad-MSCs was isolated using RNAeasy® Plus Mini Kit and reverse transcribed with RETROscript (Thermo Fisher Scientific, Waltham, USA). cDNA was subjected to quantitative real-time PCR (qPCR) using FastStart Universal SYBR Green Master master mix (Roche, Indianapolis, USA) and specific primers for differentiation-related genes and for human codon-optimized CXCR4 and IL10 sequences (Table S1). qPCRs were run on a 7500 fast real-time PCR system (Thermo Fisher Scientific, Waltham, USA). The results were normalized to human GAPDH expression and expression of control samples according to the 2^−ΔΔCt^ method.

### CXCR4 and IL10 protein expression

The expression of CXCR4 on the cell surface of Ad-MSCs was determined by flow cytometry after labeling with a phycoerythrin (PE)-conjugated anti-human CXCR4 antibody for 30 min at 4 °C (Biolegend, CA, USA). IL10 levels secreted by Ad-MSCs were measured in the supernatant of cultured cells using the human IL10 Quantikine ELISA Kit (R&D System, Minneapolis, MN, USA).

### CXCR4 western blot

Total protein extracts were isolated from Ad-MSCs using the RIPA buffer (Thermo Fisher Scientific, Waltham, USA) containing a protease inhibitor mixture (Merck, Darmstadt, Germany). Twenty micrograms of each of the cell lysates was resolved in 4–12% polyacrylamide gels (Bio-Rad, CA, USA) and transferred to the PVDF membranes (Bio-Rad, CA, USA). The membranes were blocked with 5% v/v non-fat dry milk in 0.1% Tween-20 diluted in phosphate-buffered saline (PBS; Merck, Darmstadt, Germany). Samples were immunoblotted by incubation with rabbit monoclonal anti-human CXCR4 antibody (ab124824, Abcam, Cambridge, UK) diluted in blocking solution. Rabbit anti-human vinculin (ab129002, Abcam, Cambridge, UK) was used as a loading control. Blots were visualized with Clarity Western ECL substrate (Bio-Rad, CA, USA) using a ChemiDoc MP System and ImageLab software (Bio-Rad, CA, USA).

### Immunofluorescence staining

Five thousand Ad-MSCs were plated onto 4-well Nunc™ Lab-Tek™ chamber slides (Thermo Fisher Scientific, Waltham, USA) for 48 h. Cells grown on slides were fixed with paraformaldehyde 4% for 30 min at room temperature, incubated with 0.25% Triton® X-100 (v/v) in PBS for 15 min, and washed three times with PBS. Non-specific staining sites were blocked with 2% BSA in PBS for 1 h. Cells were incubated for 1 h at room temperature with rabbit primary antibody CXCR4 (Thermo Scientific, Cat # PA3-305) in 2% BSA in PBS in a humidified chamber. Samples were washed with PBS and stained with goat anti-rabbit IgG secondary antibody, Alexa Fluor 594 conjugate (Thermo Scientific, Cat #A-11012), in 2% BSA in PBS for 1 h at room temperature in the dark. Alexa Fluor® 555 Phalloidin (Cell Signaling, USA, Cat # 8953) was used to stain the cytoskeleton. The cell nuclei were stained with nuclear binding 4,6-diamidino-2phenylindole (DAPI, D1306, Thermo Fisher Scientific, Waltham, USA) dye. Images were taken with a Zeiss Axioplan microscope (Zeiss, Jena, Germany).

### MSC harvesting and slide preparation

MSC harvesting and slide preparation were performed using standardized protocols for fibroblast cultures with modifications. Instead of the consecutive passes with Carnoy fixative, three individual washes with the following solutions were done: (1) fixation with water, 5% acetic acid at 90%, and 3% methanol; (2) methanol; and (3) Carnoy fixative.

### G-banding by trypsin and metaphase analysis

G-banding of the metaphases was done following the standardized protocols [42]. Metaphase/karyotype analyses were performed under a light microscope. All available metaphases were analyzed, and the final karyotype was reported following the European guidelines for constitutional cytogenomic analysis [43].

### Array CGH technique and analysis

DNA extraction was done with the EZ1® robot and EZ1® DNA tissue kit (Qiagen, Hilden, Germany). The automatic protocol was done following the manufacturer’s instructions. For the purpose, 1 ml of cell suspension from the culture was used. The microarray-based comparative genomic hybridation (array CGH) technique was done using the PerkinElmer/Signature Genomics 8x60K platform following the manufacturer’s protocol (CGX™ Array training guide, version 1.2, 8x60K). Data analysis and interpretation were done with the Genoglyphix® program (Genoglyphix user guide 3.0) and following the European guidelines for constitutional cytogenomic analysis.

### RNA-Seq assays

Total RNA was isolated using an RNeasy mini kit (Exiqon; Qiagen, Hilden, Germany) following the manufacturer’s protocol. RNA quality and quantity were analyzed on ND-1000 spectrophotometer (Nanodrop Technology, USA). Furthermore, RNA quality was assessed by Bioanalyser RNA 6000 kit (Agilent Technologies, CA, USA) to determine the RIN number. Only samples with RIN ≥ 7 were included for sequencing. Next-generation sequencing (NGS) libraries were constructed with the TruSeq® Stranded mRNA LT kit (Illumina, San Diego, CA) according to the manufacturer’s protocol. Bioanalyser (Agilent Technologies, CA, USA) was used for quality control of indicated steps as recommended by the manufacturer. Single-read sequencing for 75 cycles was run in an Illumina NextSeq sequencer (Illumina Inc., San Diego, USA) using the NextSeq 500 High output kit (Illumina Inc., San Diego, USA).

### Bioinformatic analysis

Reads from the fastq files were aligned to the human genome (hg19 version) using tophat v2.1.0. Read counts were extracted using htseq-count v0.5.4p3. Normalization and differential expression analysis were performed using the DESeq2 R library [44] from the Bioconductor repository. *p* values were adjusted using the Benjamini, Hochberg, and Yekutiel (BH) method to control the false discovery rate. Up- and downregulated genes were selected using a threshold of adjusted *p* value < 0.05 and fold change > 10. Functional enrichment of upregulated genes was performed using the FatiGO method [45] implemented in the Babelomics v5 suite [46] with Gene Ontology [47] and KEGG pathway annotations [48] retrieved using REST services. Significant terms were extracted using an FDR-adjusted *p* value < 0.05.

### Quantification of secreted cytokines and factors

Ad-MSCs were seeded in 6-well plates at a concentration of 1 × 10^5^ cells/well. At 4 h post-transfection, supernatants were collected, and secreted PGE_2_, TGFβ1, IL8, and IFNα were quantified by ELISA (R&D System, Minneapolis, MN, USA). Secreted IL6, IFNγ, and TNFα were quantified by flow cytometry using the *LEGENDplex™ Human Th Cytokine Panel* (Biolegend, CA, USA) following the manufacturer’s protocol.

### Cell migration assay

Migration assays were carried out in transwells with an 8-μm pore polycarbonate membrane insert (Costar, Cambridge, MA). 5 × 10^3^ Ad-MSCs were placed in the upper insert chamber of the transwell assembly. The lower chamber contained murine or human SDF-1 (Peprotech, NJ, USA) at a final concentration of 100 ng/ml. Twenty-four hours after incubation, the upper part of the membrane was scrapped gently by a cotton swab to remove non-migrating cells and washed with PBS. The membrane was fixed with 3.7–4% formalin overnight at 4 °C and stained with hematoxylin for 4 h at RT. The number of migrating cells was determined by the scoring of four random fields per well under the Nikon Eclipse E400 microscope (× 10) (Nikon, Greater London, UK), and pictures were obtained with a Leica DFC420 camera (Leica, Buckinghamshire, UK).

### In vitro immunosuppression assay

Heparinized peripheral blood samples from healthy donors were obtained from the Madrid Community Transfusion Centre under their Institutional Review Board (IRB) approval and their written informed consent and in compliance with the Helsinki Declaration.

Peripheral blood mononuclear cells (MNCs) were obtained by Ficoll-Paque PLUS (GE Healthcare Bioscience, Uppsala, Sweden) density gradient from heparinized peripheral blood samples. MNCs were marked with the intracellular fluorescent dye Carboxyfluorescein diacetate succinimidyl ester (*CellTrace™ CFSE Cell Proliferation Kit*; Molecular Probe/Invitrogen, USA), following a previously described protocol [49]. Before co-culture, Ad-MSCs were plated in 24-well plates at a concentration of 5 × 10^4^ cells/well. Twenty-four hours later, 5 × 10^5^ MNCs were added to each well in the presence of 10 μg/mL of phytohemagglutinin (PHA; Sigma Aldrich, St. Louis, MO, USA) to induce the T cell proliferation. After 3 days of incubation, cells harvested from culture wells were analyzed by flow cytometry for cell proliferation. Data were analyzed with ModFit LT™ (Verity Software House, Topsham, ME, USA).

### LPS-induced inflamed pad model

Female FVB/NJ mice (10–12 weeks old) were obtained from the Jackson Laboratory (Bar Harbor, ME) and were kept under standard pathogen-free conditions and given autoclaved food and water ad libitum in the animal facility of CIEMAT (Registration No. ES280790000183). All animal experiments were performed in compliance with the European and Spanish legislations and institutional guidelines (Spanish RD 53/2013, Law 6/2013, and European Directive 2010/63/UE). The protocol was approved by the CIEMAT Animal Experimentation Ethical Committee according to the approved biosafety and bioethics guidelines (Protocol number: PROEX 252-19).

FVB/NJ mice were sedated using isoflurane (4–5% for induction and 1–2% for maintenance) and injected subcutaneously with lipopolysaccharide (LPS) or PBS into the walking pad area (ventral aspect) according to the “Footpad Injections Guidelines in Mice and Rats” published by Institutional Animal Care and Use Committee (IACUC). Forty micrograms of LPS derived from *Escherichia coli* (0111:B4, Sigma Aldrich, St. Louis, MO, USA) diluted in 30 μL of PBS was administered into the right pad of the hind leg, using a 29-G needle with the bevel facing the skin while going in with the needle. Similarly, 30 μL of PBS was injected into the left pad, which served as a control. Twenty-four hours after LPS injection, 5 × 10^5^ mRNA- or WT-MSCs were intravenously infused through the tail vein. Quantification of pad inflammation was deduced from the increment (mm) of the thickness of the LPS-treated pad relative to the thickness of the contralateral PBS-treated pad. These measurements were performed in the walking pad area using a digital caliper (ensuring minimal compression of the pad) at different times after LPS and PBS injection, either followed or not by MSC infusion (see experimental schemes in Fig. 4a and AC). The percentage of inflammation corresponding to each experimental group in Fig. 4b, d represents the comparison between the respective inflammation achieved 24 h post-LPS and the inflammation achieved at the other time points for each mouse (% inflammation in each mouse = 100 × inflammation at each time point (mm)/inflammation at 24 h post-LPS (mm). Inflammation at this time point was considered 100% in each mouse).

At the end of the experiments, mice were sacrificed by CO_2_ inhalation. Peripheral blood cells were collected to analyze the hematological parameters using the hematology analyzer Abacus (Diatrocn, Sunrise, FL, USA). Images from LPS inflamed pads were taken 48 h after Ad-MSC infusion using iPhone 11 camera (Apple Inc., CA, USA).

### Histological and flow cytometry analysis

Pads were excised and fixed in formalin at RT before being embedded in paraffin and sectioned. To determine the changes in the tissue architecture, the sections were stained with hematoxylin and eosin (H&E). Images were obtained with a Nickon Eclipse E400 microscope and a Leica DFC420 camera. Quantification of polymorphonuclear cell infiltration was performed by flow cytometry. Pads were excised and mechanically processed and cell suspensions incubated with PE-conjugated anti-mouse CD45 antibody (Biolegend, CA, USA). Blood samples were collected and centrifuged. Human IL10 levels were analyzed in the serum with the human IL10 Quantikine ELISA Kit (R&D System, Minneapolis, MN, USA).

### MSC bio-distribution assays

In these experiments, mice pre-treated with LPS were used with the aim of investigating the biodistribution of the different types of MSCs after induction of local inflammation. WT and mRNA-transfected cells were labeled with CFSE following the manufacturer’s protocol (*CellTrace™ CFSE Cell Proliferation Kit*; Molecular Probe/Invitrogen, USA). Twenty-four hours and 48 h after Ad-MSC infusion, mice were anesthetized and perfused with PBS. The pads, lungs, liver, spleen, and bone marrow were excised. Organs were mechanically processed, and cell suspensions were analyzed by flow cytometry to detect CFSE^+^ cells.

### Statistical analysis

Statistical analyses were performed using the GraphPad Prism 7.0 software (GraphPad Software, USA). Data of in vitro tests are expressed as mean ± standard deviation (SD) and as mean ± the standard error of the mean (SEM) in in vivo tests. Normal distribution was analyzed by the Shapiro-Wilks test. One-way ANOVA followed by Tukey’s multiple comparison test was used to assess the difference between the groups. The non-parametric Kruskal-Wallis test followed by Dunn’s multiple comparison was used when data did not follow a normal distribution. In this study, *p* values < 0.05 were considered statistically significant. The significances are expressed as ****p < 0.0001, ***p < 0.001, **p < 0.01, or *p < 0.05.

## Results

### Enhanced expression of CXCR4 and IL10 in human Ad-MSCs transfected with specific mRNAs

Three different mRNAs were synthesized which carried codon-optimized sequences of the human *CXCR4* and *IL10* genes: CXCR4-mRNA, IL10-mRNA, and the bicistronic CXCR4-IL10-mRNA (Figure S1A). The synthesis of all these mRNAs was confirmed by agarose gel electrophoresis (Figure S1B).

To optimize the mRNA modification of Ad-MSCs, these cells were initially transfected with a control EGFP-mRNA (1 μg/100,000 cells) using different methods. Among these methods, mRNA transfection with Lipofectamine™ Messenger (3.75 μL/10 cm^2^ plate) showed the highest efficacy to generate EGFP^+^ MSCs, which reached values as high as 90% at 24 h post-transfection (Figure S2A). Higher concentrations of EGFP-mRNA did not enhance the efficacy of transfection (Figure S2B). Human Ad-MSCs were then transfected with 1 μg of either the monocistronic or bicistronic CXCR4 and IL10 mRNAs, using different concentrations of Lipofectamine™ Messenger. Twenty-four hours after transfection, CXCR4 expression and IL10 secretion were analyzed and compared with unmodified Ad-MSCs (WT-MSCs). As expected, levels of either CXCR4 (Figure S2C) or IL10 (Figure S2D) were always higher when 1 μg of the respective monocystronic mRNAs was used, as compared with 1 μg of the bicistronic mRNA (Figure S2C and D). Differences in the amount of Lipofectamine™ Messenger did not affect the efficacy of transfection of these mRNAs (Figure S2C and D). Analyses of cell viability showed that transfection with 1 μg of the monocistronic or the bicistronic constructs did not induce cytotoxic effects on Ad-MSCs (Figure S2E). Therefore, in all subsequent studies, 1 μg of the monocistronic or the bicistronic constructs were used.

**Fig. 1:**
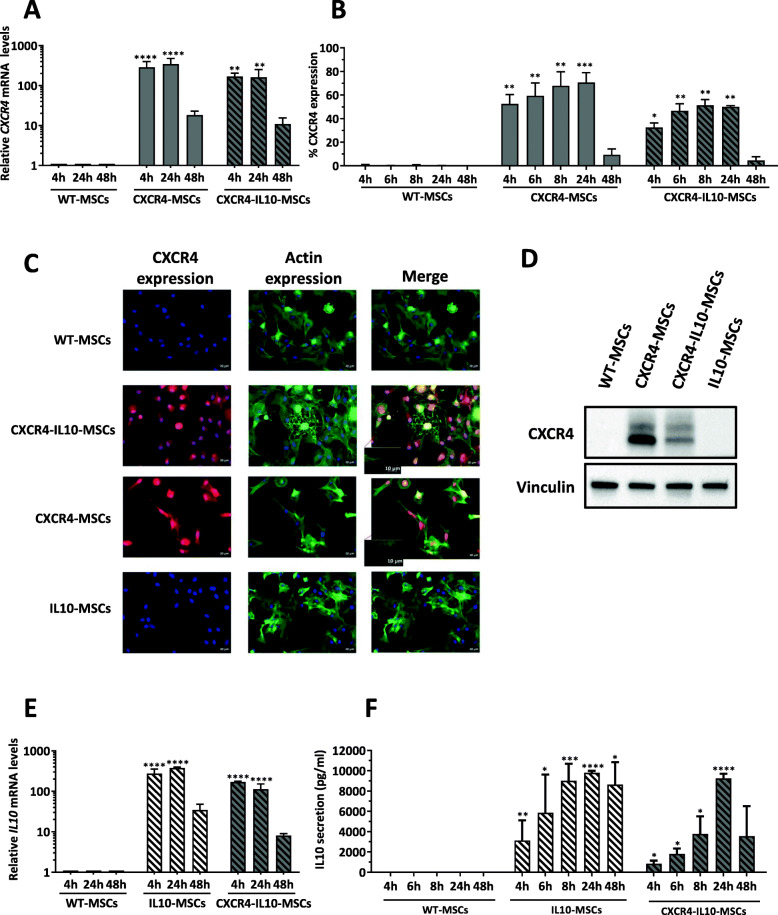
Enhanced expression of CXCR4 and IL10 in human Ad-MSCs transfected with specific mRNAs. **a** Analysis of CXCR4 mRNA levels at different times after transfection with CXCR4-mRNA and CXCR4-IL10-mRNA. **b** Flow cytometry analysis of CXCR4 in WT and mRNA-transfected Ad-MSCs at different times post-transfection. **c** Immunofluorescence staining of CXCR4 in WT and mRNA-transfected MSCs 4 h after transfection with the different mRNAs (bar = 20 μm). Inserts with higher magnifications (red frame) indicate that CXCR4-expressing MSCs resulted in the protrusion of long filopodia at the cell periphery. Bar = 10 μm. **d** CXCR4 expression analyzed by western blot 4 h after transfection with the different mRNAs using vinculin as a control. **e** Analysis of mRNA IL10 levels analyzed at different time points after transfection with IL10-mRNA and CXCR4-IL10-mRNAs. **f** IL10 secretion measured by ELISA in WT and mRNA-transfected Ad-MSCs at different time points after transfection. Bars in the plots represent mean ± SD from three independent experiments, and statistical differences were analyzed by Tukey’s multiple comparison test. *p < 0.05, **p < 0.01, ***p < 0.001, ****p < 0.0001; versus WT-MSCs

To investigate the expression of CXCR4 in Ad-MSCs transfected with either the monocistronic CXCR4-mRNA or the bicistronic CXCR4-IL10-mRNA, the levels of CXCR4-mRNA were first analyzed by qPCR at different time points post-transfection. As shown in Fig. 1a, high levels of CXCR4 expression were observed in Ad-MSCs transfected with either the monocistronic or bicistronic constructs at 4 h or 24 h post-transfection, although slightly higher levels were noted in Ad-MSCs transfected with the monocistronic construct. As expected, CXCR4-mRNA levels started to decrease at 48 h post-transfection.

To analyze the CXCR4 protein expression in these cells, flow cytometry as well as immunofluorescence and western blot analyses was conducted. As shown in the flow cytometry analyses of Fig. 1b, WT-MSCs did not express detectable levels of CXCR4 in their cell membrane. However, transfection with either the CXCR4-mRNA or CXCR4-IL10-mRNA induced a significant CXCR4 expression in the Ad-MSC membrane, as early as 4 h post-transfection (52.5 ± 15.9% and 32.5 ± 7.5% of CXCR4^+^ MSCs, respectively). As observed in the mRNA expression analyses, the proportion of CXCR4^+^ MSCs remained very high during a period of 24 h and decreased significantly at 48 h post-transfection. These analyses also showed a higher proportion of CXCR4^+^ MSCs when the monocistronic CXCR4 construct was used. The expression of CXCR4 in Ad-MSCs transfected with either the CXCR4-mRNA or the CXCR4-IL10-mRNA was confirmed by immunofluorescence (Fig. 1c) and western blot (Fig. 1d) analyses, thus revealing the efficacy of the proposed mRNA transfer to transiently express CXCR4 in Ad-MSCs.

To analyze the production of IL10 in WT and transfected Ad-MSCs, IL10-mRNA analyses were first conducted. As expected, undetectable levels of IL10-mRNA were found in WT-MSCs. As happened with CXCR4, the highest levels of IL10-mRNA were observed during the first 24 h after transfection of the monocistronic IL10 construct (Fig. 1e). ELISA analyses confirmed the rapid secretion of IL10 to the supernatant of modified Ad-MSCs, which reached levels of 9807 ± 190 pg/ml and 9262 ± 784 pg/ml in supernatants at 24 h post-transfection from Ad-MSCs transfected with the monocistronic and the bicistronic constructs, respectively (Fig. 1f). As also shown in this figure, more stable levels of IL10 secretion were noted when Ad-MSCs were transfected with the monocistronic, as compared to the bicistronic mRNA.

These results demonstrate that transfection of human Ad-MSCs with mRNAs encoding for CXCR4 and/or IL10 results in a high, fast, and transient expression of these two molecules in transfected MSCs.

### mRNA-transfected Ad-MSCs maintain the characteristic morphology, immunophenotype, differentiation capacity, and genetic stability of MSCs

**Fig. 2:**
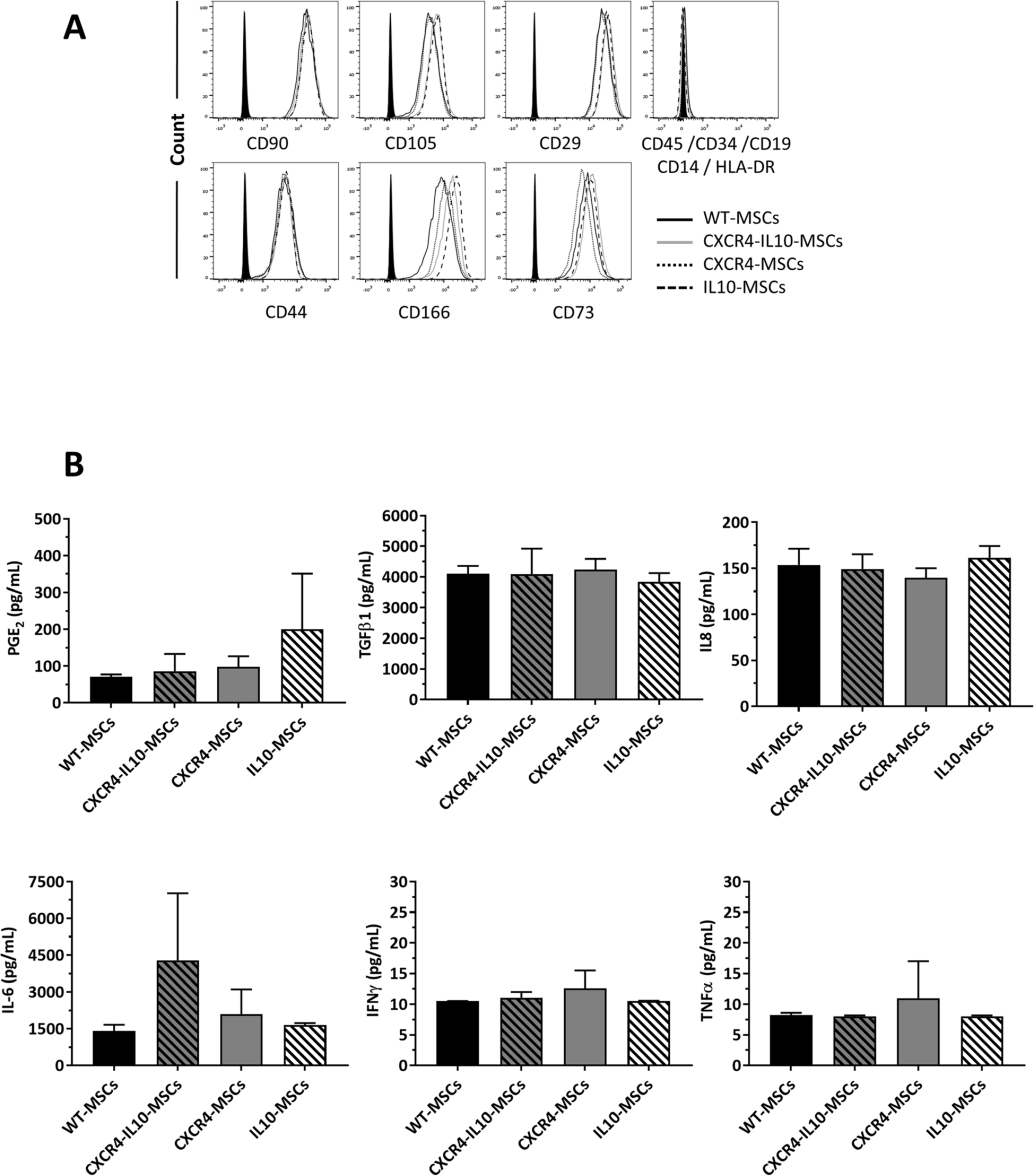
mRNA-transfected Ad-MSCs maintain the characteristic immunophenotype and secretome of WT-MSCs. **a** Immunophenotype analysis of WT-MSCs and mRNA-transfected Ad-MSCs. Representative analyses of characteristic MSC markers are shown. **b** Analysis of secreted PGE_2_, TGFβ1, IL8, IL6, IFNγ, and TNFα 4 h after transfection of Ad-MSCs with the different mRNAs. Levels of PGE_2_, IL8, and TGFβ1 were quantified by ELISA, while IL6, IFNγ, and TNFα levels were determined flow cytometry using fluorescence-encoded beads. Bars represent the mean ± SD of *n* = 3 different experiments

Next, we tested the phenotype of mRNA-transfected Ad-MSCs according to the ISCT criteria [50]. All types of Ad-MSCs displayed similar plastic adherence and characteristic spindle-shaped and fibroblastic-like morphology of WT-MSCs (Figure S3A)**.** Flow cytometry analyses showed that mRNA-transfected Ad-MSCs were negative for the expression of hematopoietic markers including CD34, CD45, CD14, CD19, and HLA-DR (≤ 5% of expression) and positive for CD90, CD44, CD105, CD166, CD29, and CD73 expression (≥ 95% of expression), in good consistency with the immunophenotype of WT-MSCs (Fig. 2a).

The alkaline phosphatase activity (Figure S3B) and the presence of lipid vacuoles (Figure S3C) after differentiation towards osteogenic and adipogenic differentiation protocols confirmed the multipotent differentiation ability of mRNA-transfected Ad-MSCs. Moreover, when the expression of differentiation-related genes was analyzed by qPCR, no differences between WT-MSCs and transfected Ad-MSCs were observed (Figure S3D and E).

Genome-wide expression analyses were also conducted in Ad-MSCs that had been transfected with the bicistronic CXCR4-IL10-mRNA. A total of 14 genes were upregulated in CXCR4-IL10-MSCs compared to WT-MSCs (adjusted *p* value < 0.05 and FC > 10, see Table S2). A functional enrichment of the upregulated genes retrieved 9 KEGG pathways that were over-represented in CXCR4-IL10-MSCs (Figure S3F and Table S3). The top 25 Gene Ontology terms over-represented in CXCR4-IL10-MSCs are shown in Figure S3G. Some of them revealed a cellular response to mRNA or viral infections, while also an over-representation of pathways related to cytokine signaling were identified. When secreted levels of different inflammation-related molecules (PGE_2_, TGFβ1, IL8, IL6, INFγ, and TNFα) were analyzed 4 h post-transfection, non-significant differences were observed among WT and the different type of transfected MSCs (Fig. 2b), apart from IL10. The transfection of MSCs with mRNAs did not change their secretion profile. Since IFNα could be induced as an innate immune reaction to mRNA transfection, levels of this cytokine were analyzed in supernatants of WT and mRNA-transfected MSCs, although no detectable levels were observed in any case (data not shown).

To test the genetic stability of mRNA-transfected Ad-MSCs, karyotype and array-CGH analyses were performed in WT and CXCR4-IL10-MSCs. No clonal abnormalities were found either in the karyotype (Figure S4A) or in copy number variants (CNV) analyses (Figure S4B), different from the ones collected in the database of genomic variants (DGV), which are considered as normal structural variations (dgv.tcag.ca/dgv/app/home).

According to these results, we can conclude that transfection of Ad-MSCs with CXCR4 and/or IL10 mRNAs does not modify the characteristic phenotype, differentiation ability, and genetic stability of Ad-MSCs.

### Increased in vitro migration and immunosuppression ability of Ad-MSCs transfected with CXCR4 and IL10 mRNAs

**Fig. 3:**
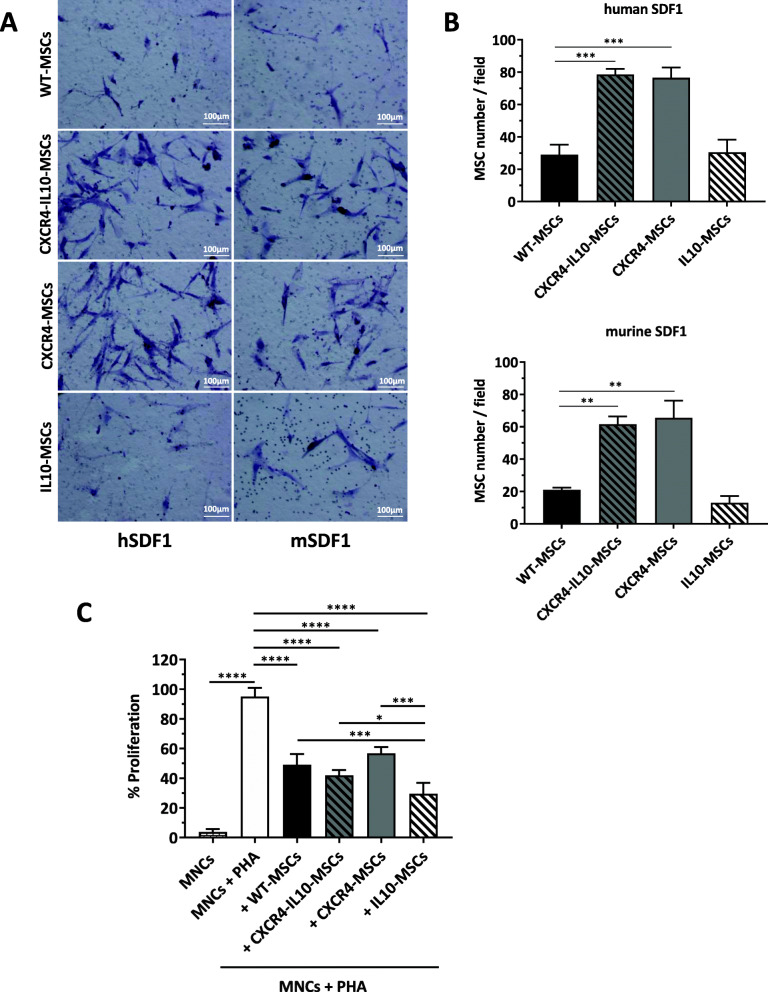
Increased in vitro migration and immunosuppression ability of Ad-MSCs transfected with CXCR4 and IL10 mRNAs. **a** Representative images of transwell-migrating WT and mRNA-transfected Ad-MSCs (4 h post-transfection) in response to human (left) or murine (right) SDF-1. Cells in the transwell membranes were stained with hematoxylin (bar = 100 μm). **b** Quantification of the number of migrated cells in response to human (graphic above) or murine (graphic bellow) SDF-1. Data were collected from three randomly chosen fields under a microscope. **c** Inhibition of lymphocyte proliferation in vitro. Human PBMCs were cultured with or without WT or mRNA-transfected MSCs in the presence of PHA for 3 days at MSC/PMBC ratios of 1/10. The proliferation of CFSE-labeled PBMCs was assessed by flow cytometry, and the effect of Ad-MSCs on PBMCs was calculated as percentage of proliferation compared with the proliferative response in PHA-stimulated lymphocytes without Ad-MSCs (positive control, white bar). Data represents the mean ± SD of at least three different experiments analyzed by Tukey’s multiple comparison test. *p < 0.05, **p < 0.01, ***p < 0.001, ****p < 0.0001

To test the migration capacity of mRNA-transfected Ad-MSCs compared to WT-MSCs, a chemotaxis assay to human and mouse SDF1α was performed. As shown in representative pictures of Fig. 3a and in the mean values of migrating Ad-MSCs included in Fig. 3b, CXCR4-MSCs and CXCR4-IL10-MSCs evidenced an enhanced migration to human or mouse SDF1α, compared to WT-MSCs (around a 3-fold increase in the number of migrated cells). As expected, Ad-MSCs transfected with IL10-mRNA showed similar migration ability to WT-MSCs.

In the next set of experiments, we studied the ability of the different Ad-MSCs to suppress the PHA-induced proliferation of human T cells. For this purpose, WT-MSCs or mRNA-transfected Ad-MSCs were co-cultured with allogeneic peripheral blood mononuclear cells (MNCs) in the presence of PHA. Three days after initiating these co-cultures, the division cycles of incubated T cells were tested by flow cytometry. As shown in Fig. 3c, PHA-stimulated T cell proliferation was significantly reduced by all types of Ad-MSCs. Nevertheless, a more marked inhibition of T cell proliferation was observed when co-cultures were established with CXCR4-IL10-MSCs and more significantly with IL10-MSCs, probably due to the higher and more stable levels of IL10 secreted by these cells, as compared to CXCR4-IL10-MSCs (see Fig. 1f).

These results show that Ad-MSCs ectopically expressing CXCR4 and/or IL10 exhibited improved in vitro migration ability and enhanced immunomodulatory properties compared to WT-MSCs.

### Enhanced in vivo anti-inflammatory and migration potential of Ad-MSCs co-expressing CXCR4 and IL10

**Fig. 4:**
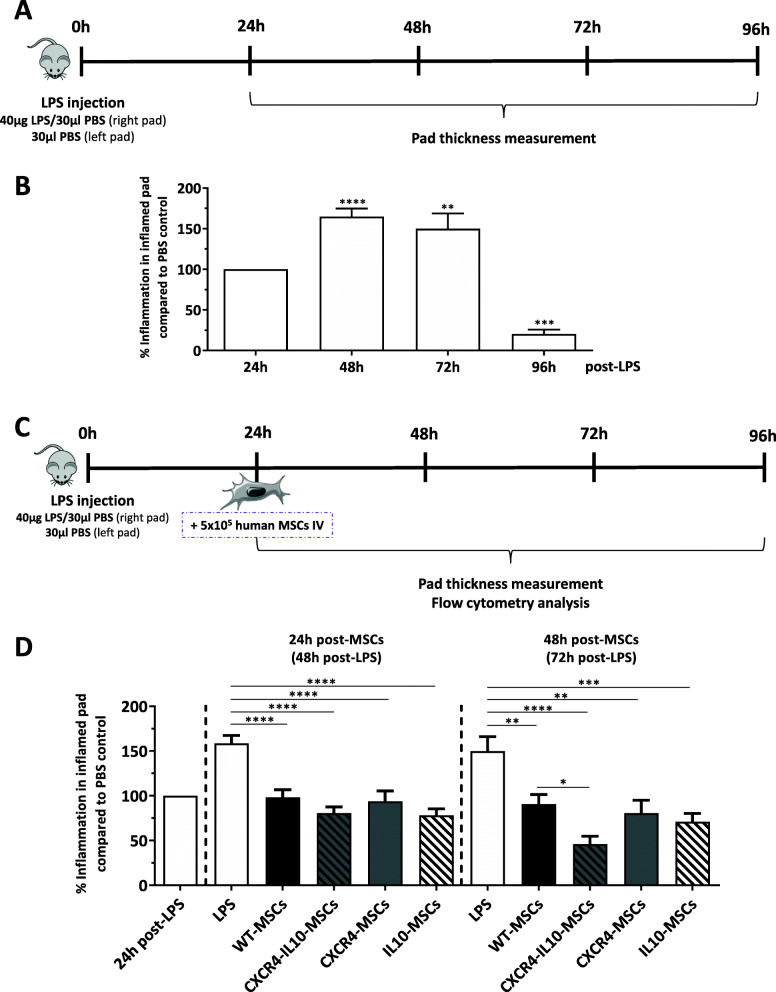
Enhanced in vivo anti-inflammatory and migration potential of Ad-MSCs co-expressing CXCR4 and IL10. **a** Schematic diagram of a local inflammation mouse model based on the injection of LPS in the right pad of mice. **b** Characterization of the LPS-induced inflammation in mouse pads. Pad thickness was determined with an electronic caliber at different time points after LPS injection. Data are represented in comparison with the thickness of the contralateral pad injected with PBS. The statistical analysis was made with respect to the 24 h post-LPS group. **c** Schematic diagram used to investigate the anti-inflammatory effects of Ad-MSCs in LPS-induced inflamed pads. **d** Evolution of local inflammation of LPS-treated pads at different time points after Ad-MSC infusion. Data are represented as the percentage of the inflammation observed in LPS-treated animals infused with WT-MSCs. Data represents the mean ± SEM of at least *n* = 15 mice/group with Tukey’s multiple comparison test applied. *p < 0.05, **p < 0.01, ***p < 0.001, ****p < 0.0001

To test the anti-inflammatory properties of mRNA-transfected Ad-MSCs, in vivo experiments were performed in mice in which a local inflammation was induced by means of the injection of 40 μg LPS into the right pad, and an equivalent volume of PBS into the left pad, as control (Fig. 4a). First, we characterized the kinetics of pad inflammation as well as changes in the number of peripheral blood leukocytes in mice that only received LPS. As shown in Fig. 4b, a transient local inflammation in treated pads was observed after LPS administration, which was resolved by the fourth day post-LPS administration. This effect was associated with a transient decrease in the number of circulating white blood cells (WBCs) (Figure S5A) due to a marked decrease in the number of lymphocytes (Figure S5B) compared to the untreated control group.

After establishing the LPS mouse model, we studied the effect that the different types of Ad-MSCs had on pad inflammation. One day after LPS injection, 5 × 10^5^ WT or mRNA-transfected Ad-MSCs were intravenously infused 4 h after transfection. At this time, CXCR4 levels were already significant, and the expression of this transgene and of IL10 in vivo should remain high for at least 24 h (see Fig. 1). Compared to the LPS-control group, all animals that received Ad-MSCs showed a significant reduction in the pad inflammation at 24 h post-MSC infusion. Nevertheless, analyses conducted 24 h later (48 h post-MSC infusion) revealed that the inflammation observed in mice infused with CXCR4-IL10-MSCs was significantly lower compared to mice treated with WT-MSCs (46.05 ± 8.74% versus 90.82 ± 10.58%, respectively (Fig. 4d). Moreover, these studies indicated that in contrast to any other condition, inflammation in pads of mice treated CXCR4-IL10-MSCs was almost completely resolved at 48 h post-therapy. No differences in the number of white blood cells, red blood cells, or platelets were observed among non-MSC-treated and any type of MSC-treated groups of mice (Figure S5C).

**Fig. 5:**
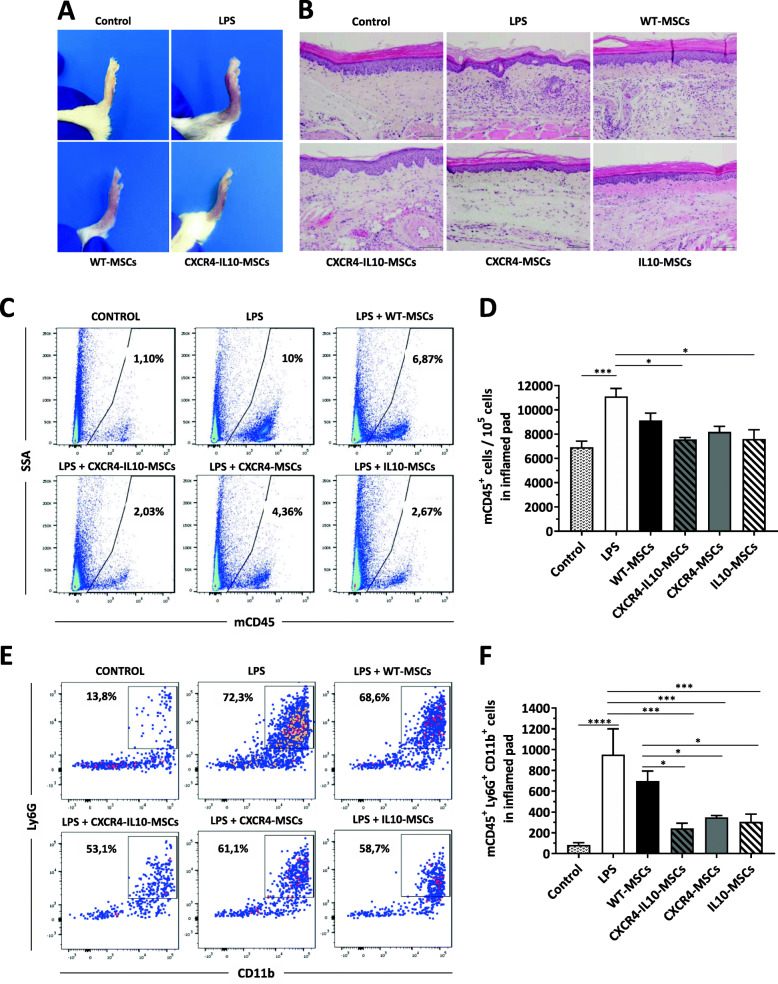
Reduced neutrophil infiltration in inflamed pads in mice treated with Ad-MSCs expressing CXCR4 and/or IL10 compared to WT-MSCs. **a** Representative images of inflamed pads from LPS and Ad-MSC treated mice 48 h after infusion of WT or mRNA-transfected MSCs. **b** Representative histological images of inflamed pads 48 h after injection of WT or mRNA transfected MCSs. Five sections/pad were stained with hematoxylin and eosin (bar = 200 μm). **c** Representative flow cytometry histograms showing the proportion of murine CD45^+^ cells in inflamed pads from mice treated with WT and mRNA-transfected MSCs. **d** Quantification of the flow cytometry analysis of CD45^+^ cells shown in **b**. **e** Representative flow cytometry analysis of neutrophils (Ly6C^−^ Ly6G^+^ CD11b^+^ cells) in CD45^+^ cells infiltrating the inflamed pads from mice treated with WT and mRNA-transfected MSCs. **f** Quantitation of the flow cytometry analysis of neutrophil infiltration in the inflamed pads from mice treated with WT and mRNA-transfected MSCs. Bars represent the mean ± SEM of at least *n* = 3 mice/group, and statistical significance was determined using Tukey’s multiple comparisons test. *p < 0.05, **p < 0.01, ***p < 0.001

Figure 5a shows representative pictures of pads from PBS (control) and LPS-treated pads, from mice that received or not MSCs (WT and CXCR4-IL10 MSCs). When the tissue architecture was studied (see representative pictures in Fig. 5b), PBS-injected pads showed normal tissue architecture, while LPS-injected pads revealed a substantial alteration of the pad’s architecture. Figure 5b also shows an evident leukocyte infiltration in LPS-treated pads, while this was much more limited after infusion of WT or mRNA-transfected MSCs. To quantify the level of leukocyte infiltration in the different experimental groups, flow cytometry analyses were performed 48 h after MSC infusion. These analyses showed that CD45^+^ cell infiltration observed in LPS-treated pads was significantly reduced when either CXCR4-IL10-MSCs (7566 ± 156 CD45^+^ cells/10^5^ cells in inflamed pad) or IL10-MSCs (7597 ± 767 CD45^+^ cells/10^5^ cells in inflamed pad) were infused, as compared to the LPS group (11,112 ± 657 CD45^+^ cells/10^5^ cells in inflamed pad; Fig. 5c, d). As shown in Fig. 5e, this cell infiltration was mainly due to neutrophils, the first cell type that responds to an inflammatory stimulus. A marked reduction in the number of infiltrating neutrophils was observed in pads from mice treated with any of the mRNA-modified Ad-MSCs, compared to WT-MSCs (Fig. 5f).

Serum levels of human IL10 were also analyzed by ELISA 24 and 48 h after MSC infusion. Significantly, only detectable levels of this cytokine were noted in mice treated with CXCR4-IL10-MSCs and more markedly in IL10-MSC-treated mice (Figure S6A). Moreover, non-significant differences were observed when mouse IFNγ, TNFα, IL1β, and IL6 were analyzed in the sera from the different mouse groups (Figure S6B).

**Fig. 6:**
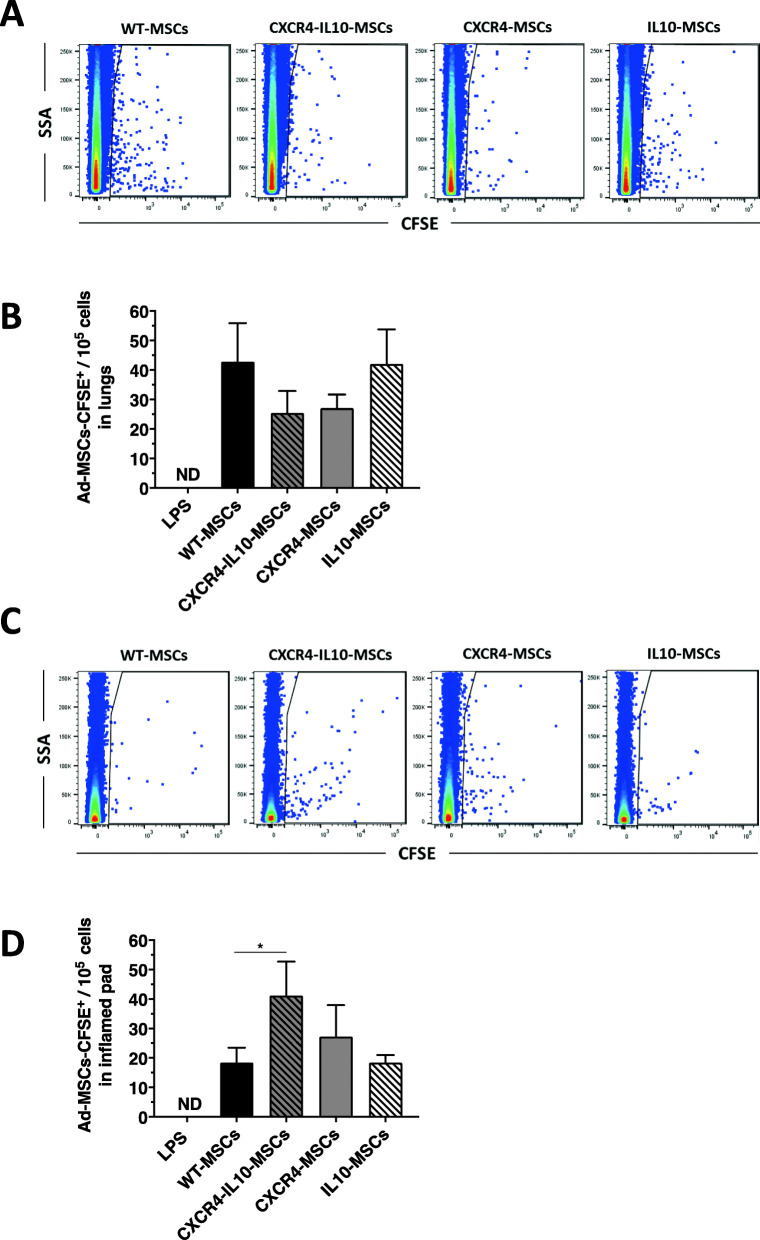
Enhanced homing of Ad-MSCs co-expressing CXCR4 and IL10 into the inflamed pads. **a** Representative flow cytometry analyses of CFSE-labeled Ad-MSCs in the lungs from mice pre-treated with LPS (local administration in one pad) and infused with WT or mRNA-transfected MSCs. **b** Quantification of the presence of Ad-MSCs-CFSE^+^ in the lungs. **c** Representative flow cytometry analyses of CFSE-labeled Ad-MSCs in the inflamed pads from mice pre-treated with LPS (local administration in one pad) and infused with WT or mRNA-transfected MSCs. **d** Quantification of the presence of Ad-MSCs-CFSE^+^ in the inflamed pads. Analyses were conducted 24 h after the infusion of Ad-MSCs. Bars represent the mean ± SEM of at least *n* = 10 mice/group using Dunn’s multiple comparison test. *p < 0.05; versus WT-MSCs

Finally, we also evaluated the presence of WT or mRNA-modified Ad-MSCs in different tissues of LPS-treated mice. To facilitate the identification of Ad-MSCs in the different mice tissues, Ad-MSCs were labeled with a vital dye (CFSE) prior to injection. When these analyses were conducted at 24 h post-Ad-MSC infusion, detectable numbers of Ad-MSCs-CFSE^+^ were observed in the lungs. Interestingly, when mice were infused with CXCR4-MSCs or CXCR4-IL10-MSCs, the numbers of CFSE^+^ cells trapped in the lungs were lower than those determined in any of the other groups (Fig. 6a, b), although differences did not reach statistical significance. The presence of CFSE-labeled MSCs was also investigated in the BM of these mice, since this is a tissue expressing high levels of SDF1 [51]. While low numbers of WT-MSCs were detected, these numbers were significantly increased in mice infused with CXCR4-Il10 MSCs (Figure S7).

Remarkably, when Ad-MSCs were investigated in inflamed pads, significantly higher numbers were observed in mice infused with CXCR4-IL10-MSCs compared to the WT-MSC group (Fig. 6c, d), revealing the enhanced migration of these cells to this inflamed tissue. Moreover, the concentration of CXCR4-IL10-MSCs in inflamed pads was higher than that observed either in lungs or BM (see Figs. 6 and S7). Flow cytometry analyses conducted 48 h after infusion of any type of Ad-MSCs did not detect MSCs in the lungs, liver, spleen, lymph nodes, PBS-treated pads, or LPS-treated pads. This observation was confirmed by q-PCR using a specific probe for human albumin (data not shown).

This final set of in vivo studies demonstrated that the transient ectopic expression of CXCR4 and IL10 in Ad-MSCs enhances their migration to inflamed sites and improves the anti-inflammatory effects of Ad-MSCs in a mouse model of local inflammation.

## Discussion

Mesenchymal stromal cells have been used in more than 900 clinical trials (www.clinicaltrials.gov), both in regenerative medicine and for the treatment of inflammatory and autoimmune disorders [52]. Although these cells have shown evidence of therapeutic benefit in different preclinical and early-phase clinical studies, more advanced trials have revealed difficulties to demonstrate significant advantages of MSC therapies over standard ones, indicating the convenience of enhancing the therapeutic efficacy of these cells.

Most of the approaches used so far to genetically modify human stem cells have employed viral vectors due to the high transduction efficacy of these systems [53]. However, in those cases in which the stable expression of a specific protein is not necessary to boost the therapeutic efficacy of the target cells, the use of non-viral transient expression systems constitutes simpler and less expensive approaches, as compared to viral vectors. In particular, mRNA transfection has been successfully used not only in preclinical, but also in clinical studies for the generation of dendritic cell vaccines [54, 55]. In the case of MSCs, these cells have been engineered with up to three different mRNAs to potentiate their migration and anti-inflammatory properties in inflamed tissues [36].

Based on these premises, we investigated the efficacy of a similar mRNA transfection approach to facilitate the transient expression of two different molecules in human Ad-MSCs: CXCR4, a chemokine receptor involved in cell migration in response to SDF1 [27] and IL10, a potent anti-inflammatory cytokine [56].

In particular, we developed monocistronic as well as bicistronic CXCR4-IL10-codon-optimized mRNAs to transfect Ad-MSCs. This bicistronic mRNA construct facilitates the ectopic co-expression of two different transgenes after a single transfection process, in contrast to other approaches in which three independent mRNAs were used to co-transfect MSCs [36].

Our studies demonstrate that in contrast to WT-MSCs, which did not express detectable amounts of CXCR4 or IL10, Ad-MSCs transfected with the respective mRNAs expressed significant levels of these two molecules in a time-dependent manner. Importantly, the transient modification of Ad-MSCs with mRNAs did not alter their immunophenotype, nor their differentiation capacity or genetic stability, compared to WT-MSCs. Moreover, mRNA transfection of MSCs maintained the cytokine profile secretion of these cells and did not increase IFNα levels. These results are consistent with the previous published studies [57, 58] and suggested that under our experimental conditions, mRNA transfection did not induce an innate immune reaction in MSCs.

Moreover, our in vitro studies showed that transfection of MSCs with CXCR4 mRNAs induced an enhanced migration of these cells towards SDF-1 compared to WT-MSCs. Similarly, Ad-MSCs transfected with IL10-mRNA mediated a more significant inhibition of T cell proliferation compared to WT-MSCs.

Using an in vivo model of acute inflammation, we also demonstrated that all types of Ad-MSCs were able to exhibit anti-inflammatory effects. It is remarkable that in the particular case of CXCR4-IL10-MSCs, a more potent anti-inflammatory action was observed compared to WT-MSCs. It is also of significance that although IL10-MSCs induced more stable levels of IL10 both in vitro and also in vivo and also conferred a more marked inhibition of T cell proliferation in vitro compared to CXCR4-IL10-MSCs, MSCs transfected with the bi-cistronic mRNA were the ones that conferred superior anti-inflammatory effects in vivo. These results strongly suggest a synergistic therapeutic effect in MSCs mediated by the co-expression of CXCR4 and IL10.

Analyses of the biodistribution after systemic infusion of the different types of MSCs showed that the proportion of Ad-MSCs trapped in the lungs was moderately lower in mice infused with CXCR4-expressing Ad-MSCs. Taking into account that the lungs are the organs that trap the majority of systemically infused MSCs [59, 60], our data suggest that a proportion of CXCR4-expressing Ad-MSCs overcome the main barrier associated with the intravenous infusion of these cells. Moreover, as compared to WT-MSCs, significantly higher numbers of CXCR4-IL10-MSCs were found in inflamed pads, as well as in the bone marrow (BM), a tissue typically expressing high concentrations of SDF1 [51]. This latter observation is consistent with previous studies in which the overexpression of CXCR4 in MSCs improved MSC engraftment in BM and accelerated the hematopoietic recovery of transplanted mice [61]. The fact that in our in vivo experiments only CXCR4-IL10-MSCs showed a significantly enhanced migration to inflamed pads compared to WT MSCs suggests that the anti-inflammatory effects of IL10 may also facilitate the migration of these cells to the inflamed tissue. These results reinforce the relevance of the co-expression of CXCR4 and IL10 in MSCs, both to enhance the migration and also to mediate anti-inflammatory effects in inflamed tissues. The fact that no detectable numbers of any of the MSC types could be identified in inflamed pads or other tissues 48 h after the infusion is also consistent with other studies showing that MSCs are rapidly removed from the body [29, 62], thus exerting a transient mechanism of action [63].

## Conclusions

Taken together, our results show the improved therapeutic efficacy of human Ad-MSCs transfected with mRNAs encoding for specific migration and anti-inflammatory molecules. In particular, our data demonstrate that the transient co-expression of CXCR4 and IL10 in Ad-MSCs increases the migration of these cells to inflamed sites and enhances the anti-inflammatory properties of MSCs in an LPS-induced inflamed pad model. The proposed modification of MSCs may constitute a new step in the development of more efficient cell therapies for the treatment of inflammatory diseases.

## Supplementary Information

**Additional file 1: Figure S1.** In vitro synthesis of CXCR4-mRNA, IL10-mRNA and CXCR4-IL10-mRNA with codon-optimized human sequences. **(A)** Plasmids containing codon-optimized versions of human CXCR4 and IL10 and bicistronic coCXCR4-IL10 cDNAs for the synthesis of monocistronic and bi-cistronic mRNAs. **(B)** In vitro transcribed CXCR4-IL10-mRNA, CXCR4-mRNA and IL10-mRNA visualized on an agarose gel before and after polyA tailing and purification.

**Additional file 2: Figure S2.** Optimization of mRNA transfection in human Ad-MSCs. **(A)** Influence of different lipofectamines upon the transfection of Ad-MSCs with a control mRNA encoding for the EGFP marker protein. Transfection efficacy was analyzed by flow cytometry 24 h after transfection. **(B)** Influence of the mRNA concentration upon the transfection of Ad-MSCs. Analyses were performed as in panel A. **(C)** Analysis of human CXCR4 expression in Ad-MSCs by flow cytometry 24 h after transfection with 1 μg of CXCR4-mRNA and CXCR4-IL10 mRNA using different lipofectamine concentrations. **(D)** Human IL10 secretion of Ad-MSCs transfected with 1 μg of IL10-mRNA and CXCR4-IL10 mRNA. Analyses were performed by ELISA in supernatants collected 24h after transfection of the different mRNAs using different concentrations of lipofectamine. **(E)** Viability of Ad-MSCs analyzed at different times after transfection with the different mRNAs, using the ATP luminescence assay (see Materials and Methods). Data represents the mean ± SD of at least *n* = 3 different experiments. Statistical differences between mRNA-transfected MSCs and WT-MSCs were calculated with Tukey’s multiple comparisons test. *p < 0.05, **p < 0.01, ***p < 0.001, ****p<0.0001, versus WT-MSCs.

**Additional file 3: Figure S3.** Morphology, differentiation capacity and genome-wide expression analyses of Ad-MSCs transfected with CXCR4-IL10 mRNAs. **(A)** Plastic adherence and characteristic spindle-shaped and fibroblastic-like morphology of WT and mRNA-transfected Ad-MSCs observed by light microscopy (Bar = 100 μm). **(B)** In vitro osteogenic differentiation of WT and mRNA-transfected Ad-MSCs analyzed 10 days after incubation with specific osteogenic differentiation medium. Alkaline phosphatase deposits were observed by light microscopy after staining with Fast BCIP/NCP (Bar = 100μm). **(C)** In vitro adipogenic differentiation of WT and mRNA-transfected Ad-MSCs after 21 days of culture with the specific adipogenic differentiation medium. Lipid droplets were noted by light microscopy (Bar=100μm). **(D)** Gene expression of the osteogenic alkaline phosphatase (ALPL) and osteocalcin (bone gamma-carboxyglutamic acid-containing protein; BGLAP) markers determined by RT-qPCR. **(E)** Gene expression of the adipogenic peroxisome proliferator-activated receptor gamma (PPARG) marker quantified by RT-qPCR. **(F)** KEGG pathways over-represented in CXCR4-IL10-mRNA transfected Ad-MSCs compared to WT-MSCs. **(G)** Gene Ontology top 25 biological processes over-represented in CXCR4-IL10-MSCs compared to WT-MSCs. Terms are sorted by –log (FDR). The color intensity indicates the percentage of genes annotated with a particular term in the list of up-regulated genes.

**Additional file 4: Figure S4.** Chromosomal stability studies in WT and CXCR4-IL10-mRNA transfected Ad-MSCs. **(A**) Representative cytogenetic analysis in WT and CXCR4-IL10-transfected Ad-MSCs conducted 4 h after transfection. **(B)** Representative array CGH analysis in the same samples analyzed in panel A.

**Additional file 5: Figure S5.** Peripheral blood cell counts in mice locally infused with LPS. **(A)** Evolution of circulating white blood cell (WBCs) counts in mice injected with PBS (control group) or LPS. **(B)** Evolution of circulating lymphocytes, monocytes and granulocytes after LPS injection compared to control mice**. (C)** Counts of circulating white blood cells (WBCs), red blood cells (RBCs) and platelets 72 h after LPS injection compared to levels obtained in the LPS group not receiving MSCs. Data represent the mean ± SEM of at least *n* = 6 mice/group. Statistical differences between LPS-injected and control mice were calculated with Tukey’s multiple comparisons test. *p < 0.05, **p < 0.01, ***p < 0.001, versus control group without LPS injection (A and B) or LPS group without MSCs (C).

**Additional file 6: Figure S6.** Analysis of circulating human IL10 levels in the serum of mice treated with LPS and WT or mRNA-transfected Ad-MSCs. **(A)** ELISA analysis of circulating human IL10 levels in the serum of mice treated with LPS and WT and mRNA-transfected MSCs. Grey and white symbols represent, respectively, analyses performed 24 or 48h after MSC infusion. **(B)** Levels of circulating murine IFNγ, TNFα, IL1β and IL6 in the serum of mice treated with LPS and WT and mRNA-transfected MSCs. Differences between mice receiving only LPS and mice treated with LPS plus the different Ad-MSC groups were analyzed with Tukey’s multiple comparisons test. *p < 0.05.

**Additional file 7: Figure S7.** Enhanced response of Ad-MSCs co-expressing CXCR4 and IL10 to SDF1 in bone marrow of LPS-treated mice. **(A)** Flow cytometry analyses of CFSE-labelled Ad-MSCs in BM from mice pre-treated with LPS (local administration in one pad) and infused with WT or CXCR4-IL10-MSCs. **(B)** Quantification of the presence of Ad-MSCs-CFSE^+^ in BM. Analyses were conducted 24 hours after the infusion of Ad-MSCs. Bars represent the mean ± SEM of *n* = 3-5 mice/group. Unpaired t test was used to compare CFSE^+^ cell number in BM of mice receiving WT-MSCs with BM of those receiving CXCR4-IL10-MSCs.

**Additional file 8: Table S1.** Primers used in the different qPCRs.

**Additional file 9: Table S2.** List of up-regulated genes in mRNA-transfected Ad-MSCs compared to WT-MSCs.

**Additional file 10: Table S3.** List of up-regulated genes retrieving 9 KEGG pathways in mRNA-transfected Ad-MSCs compared to WT-MSCs.

## Data Availability

The datasets used and/or analyzed during the current study are available from the corresponding authors on reasonable request.

## Abbreviations

Ad-MSCs: Adipose tissue-derived mesenchymal stromal cells
ɑ-MEM: Minimum Essential Medium α
Array CGH: Microarray-based comparative genomic hybridation
bFGF: Human basic fibroblast growth factor
CFSE: Carboxyfluorescein diacetate succinimidyl ester
CNV: Copy number variants
CXCR4: C-X-C motif chemokine receptor type 4
DGV: Database of Genomic Variants
EGFP: Enhanced green fluorescent protein
GVHD: Graft versus host disease
H&E: Hematoxylin and eosin
HBB: Human β-globin
IL: Interleukin
LPS: Lipopolysaccharide
MNCs: Peripheral blood mononuclear cells
mRNA: Messenger RNA
MSCs: Mesenchymal stromal cells
NGS: Next-generation sequencing
PBS: Phosphate-buffered saline
PE: Phycoerythrin
PHA: Phytohemagglutinin
qPCR: Quantitative real-time PCR
SD: Standard deviation
SDF1α: Stromal cell-derived factor 1α
SEM: Standard error of the mean
UTRs: Untranslated regions
WBCs: White blood cells
WT-MSCs: Wild-type or unmodified Ad-MSCs
