# Supplementary figures and images for: Enhanced anti-inflammatory effects of mesenchymal stromal cells mediated by the transient ectopic expression of CXCR4 and IL10
Source: Stem Cell Res Ther. 2021 Feb 12;12:124. doi: 10.1186/s13287-021-02193-0 (PMC7881581; doi:10.1186/s13287-021-02193-0)

**Figure S1**

**A**

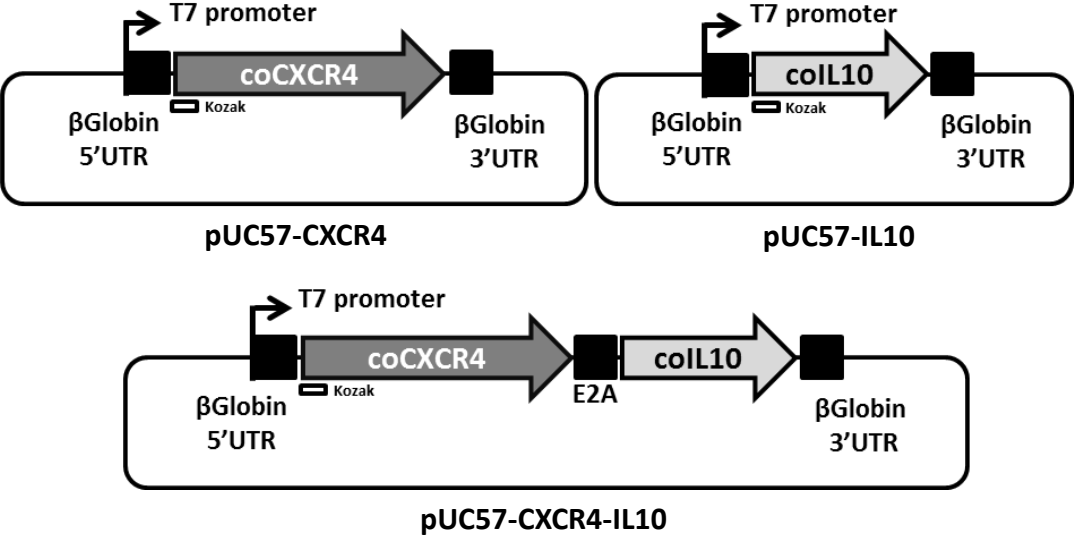

**B**

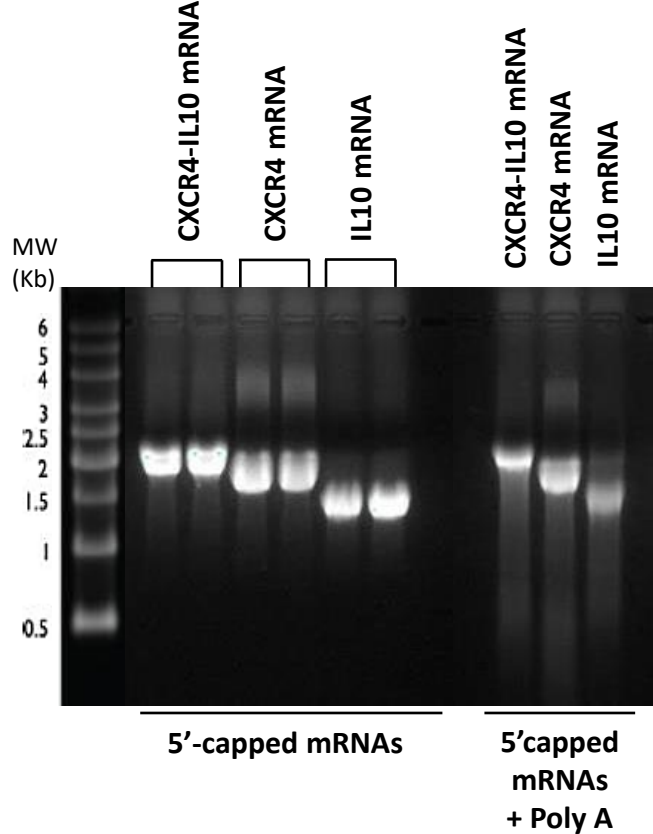

Supplement: Supplementary file 1 — Additional file 1: Figure S1. In vitro synthesis of CXCR4-mRNA, IL10-mRNA and CXCR4-IL10-mRNA with codon-optimized human sequences. (A) Plasmids containing codon-optimized versions of human CXCR4 and IL10 and bicistronic coCXCR4-IL10 cDNAs for the synthesis of monocistronic and bi-cistronic mRNAs. (B) In vitro transcribed CXCR4-IL10-mRNA, CXCR4-mRNA and IL10-mRNA visualized on an agarose gel before and after polyA tailing and purification. [file 13287_2021_2193_MOESM1_ESM.pdf]

Figure S2

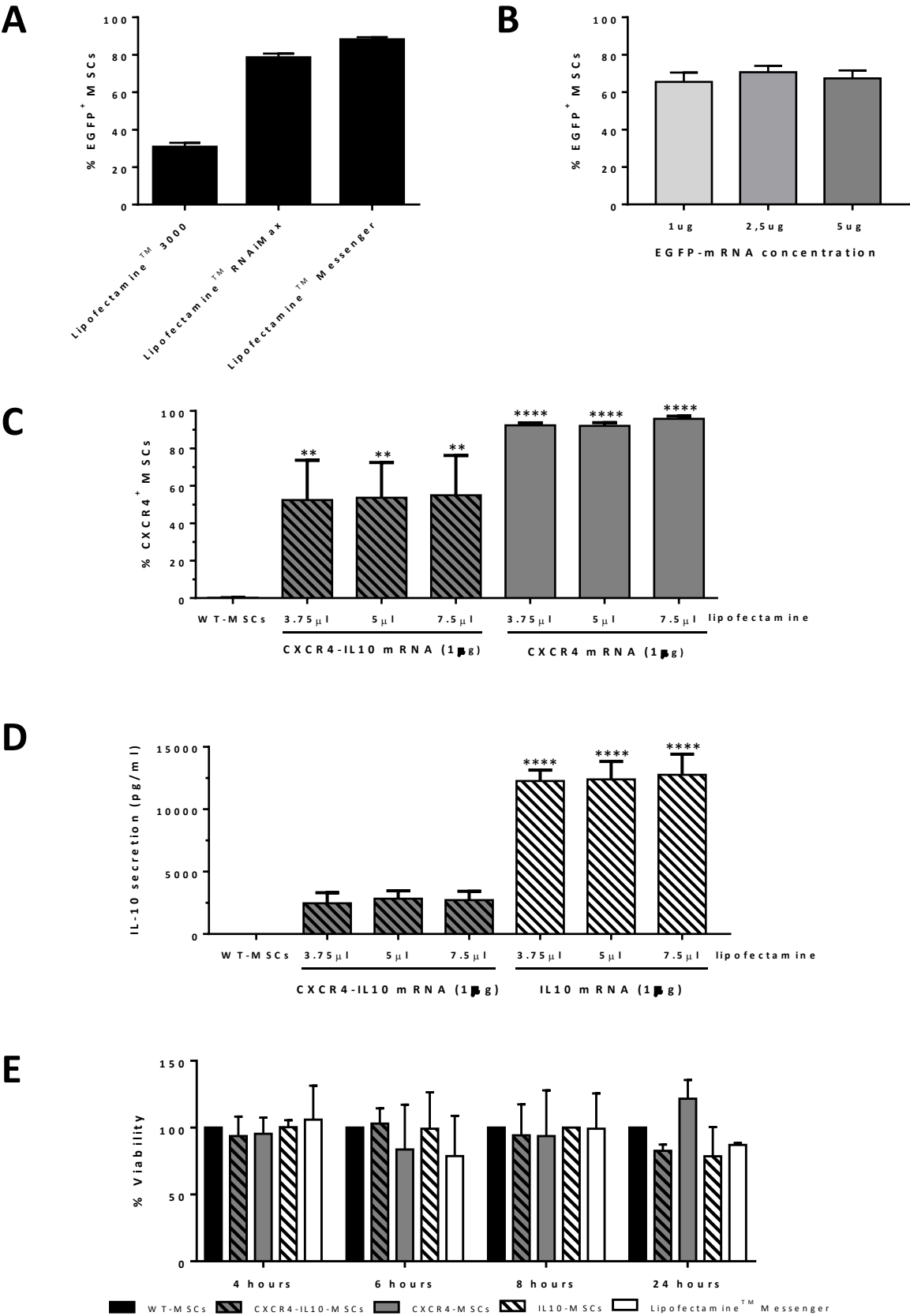

Supplement: Supplementary file 2 — Additional file 2: Figure S2. Optimization of mRNA transfection in human Ad-MSCs. (A) Influence of different lipofectamines upon the transfection of Ad-MSCs with a control mRNA encoding for the EGFP marker protein. Transfection efficacy was analyzed by flow cytometry 24 h after transfection. (B) Influence of the mRNA concentration upon the transfection of Ad-MSCs. Analyses were performed as in panel A. (C) Analysis of human CXCR4 expression in Ad-MSCs by flow cytometry 24 h after transfection with 1 μg of CXCR4-mRNA and CXCR4-IL10 mRNA using different lipofectamine concentrations. (D) Human IL10 secretion of Ad-MSCs transfected with 1 μg of IL10-mRNA and CXCR4-IL10 mRNA. Analyses were performed by ELISA in supernatants collected 24h after transfection of the different mRNAs using different concentrations of lipofectamine. (E) Viability of Ad-MSCs analyzed at different times after transfection with the different mRNAs, using the ATP luminescence assay (see Materials and Methods). Data represents the mean ± SD of at least n = 3 different experiments. Statistical differences between mRNA-transfected MSCs and WT-MSCs were calculated with Tukey’s multiple comparisons test. *p < 0.05, **p < 0.01, ***p < 0.001, ****p<0.0001, versus WT-MSCs. [file 13287_2021_2193_MOESM2_ESM.pdf]

Figure S3

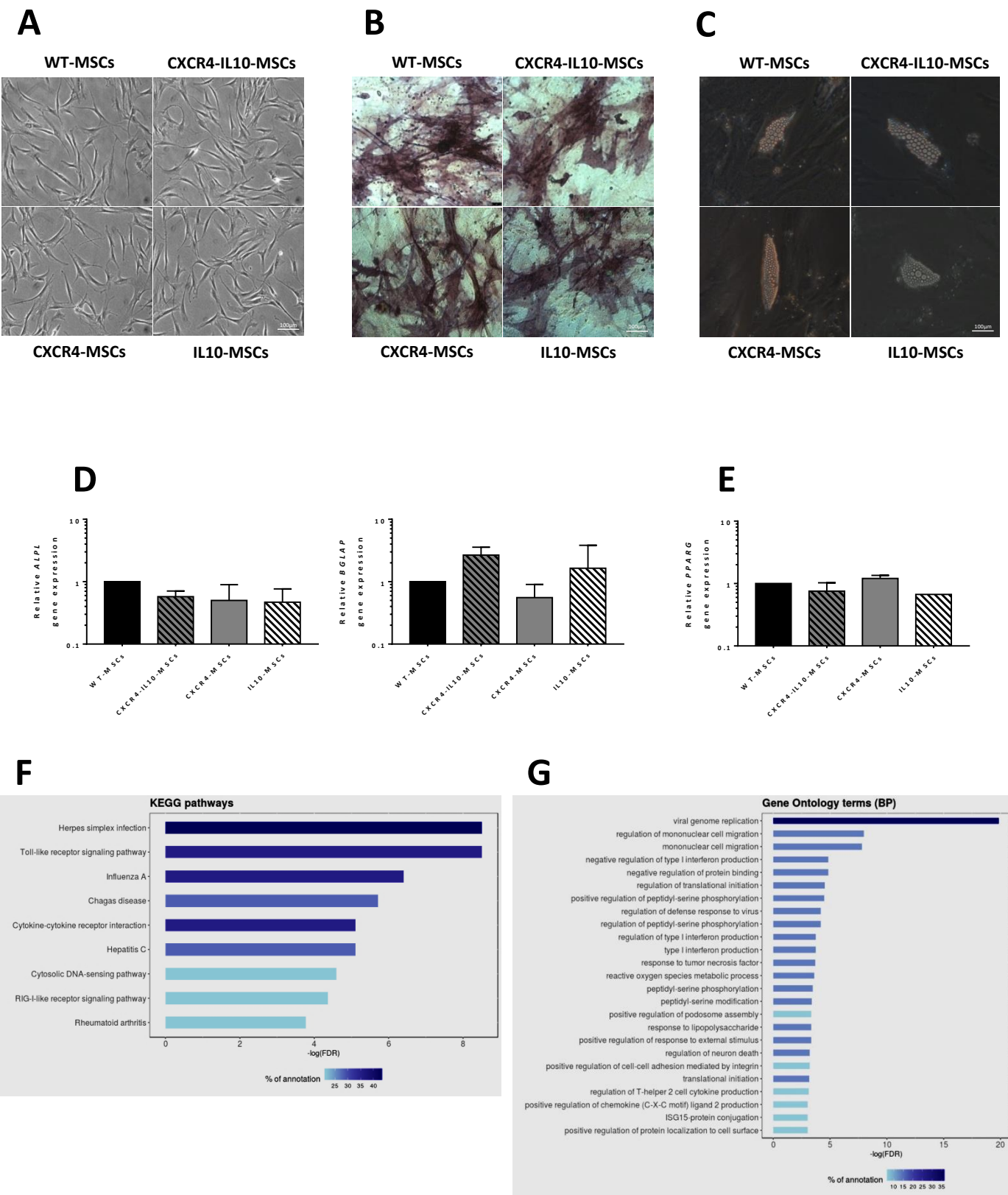

Supplement: Supplementary file 3 — Additional file 3: Figure S3. Morphology, differentiation capacity and genome-wide expression analyses of Ad-MSCs transfected with CXCR4-IL10 mRNAs. (A) Plastic adherence and characteristic spindle-shaped and fibroblastic-like morphology of WT and mRNA-transfected Ad-MSCs observed by light microscopy (Bar = 100 μm). (B) In vitro osteogenic differentiation of WT and mRNA-transfected Ad-MSCs analyzed 10 days after incubation with specific osteogenic differentiation medium. Alkaline phosphatase deposits were observed by light microscopy after staining with Fast BCIP/NCP (Bar = 100μm). (C) In vitro adipogenic differentiation of WT and mRNA-transfected Ad-MSCs after 21 days of culture with the specific adipogenic differentiation medium. Lipid droplets were noted by light microscopy (Bar=100μm). (D) Gene expression of the osteogenic alkaline phosphatase (ALPL) and osteocalcin (bone gamma-carboxyglutamic acid-containing protein; BGLAP) markers determined by RT-qPCR. (E) Gene expression of the adipogenic peroxisome proliferator-activated receptor gamma (PPARG) marker quantified by RT-qPCR. (F) KEGG pathways over-represented in CXCR4-IL10-mRNA transfected Ad-MSCs compared to WT-MSCs. (G) Gene Ontology top 25 biological processes over-represented in CXCR4-IL10-MSCs compared to WT-MSCs. Terms are sorted by –log (FDR). The color intensity indicates the percentage of genes annotated with a particular term in the list of up-regulated genes. [file 13287_2021_2193_MOESM3_ESM.pdf]

**Figure S4**

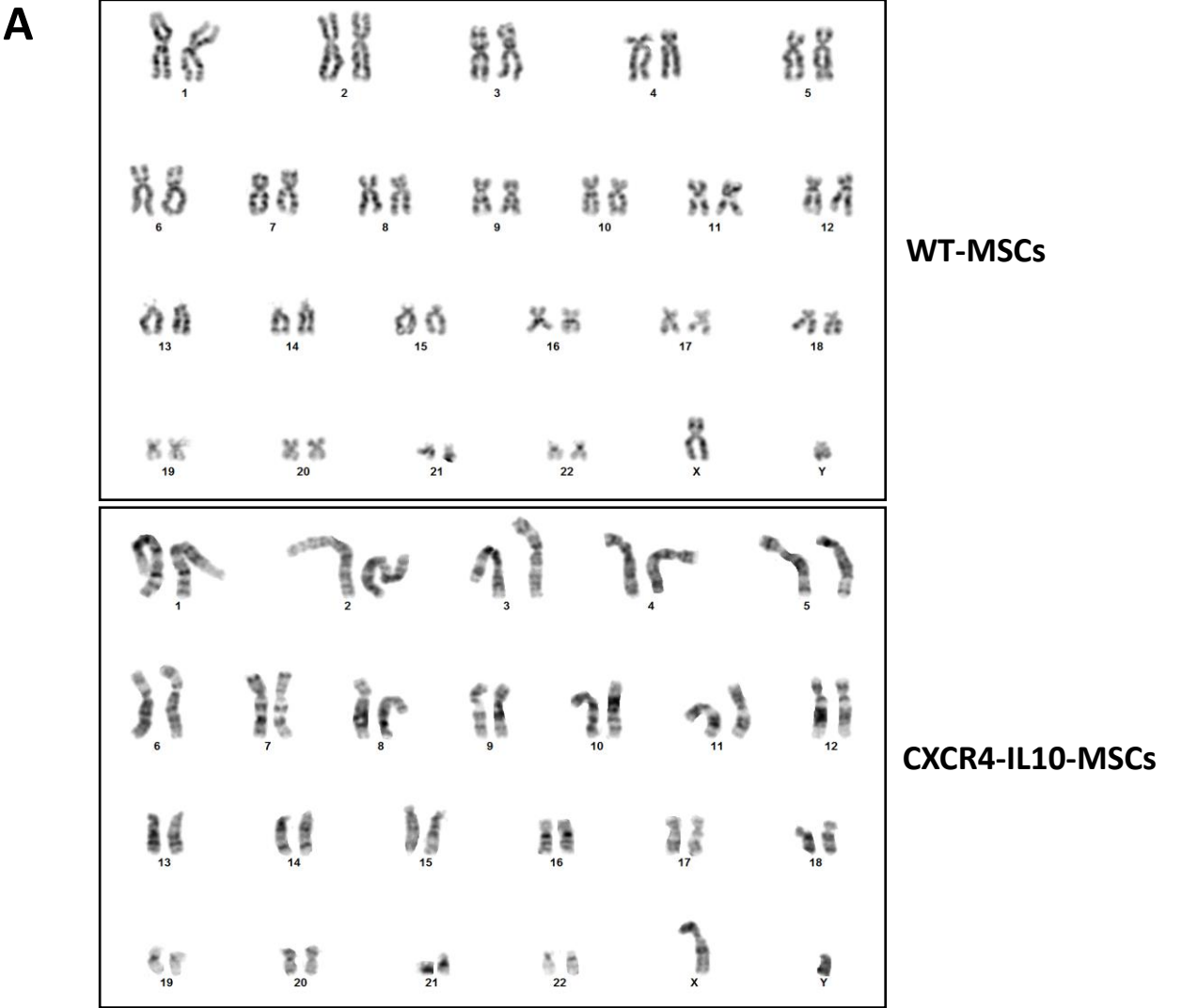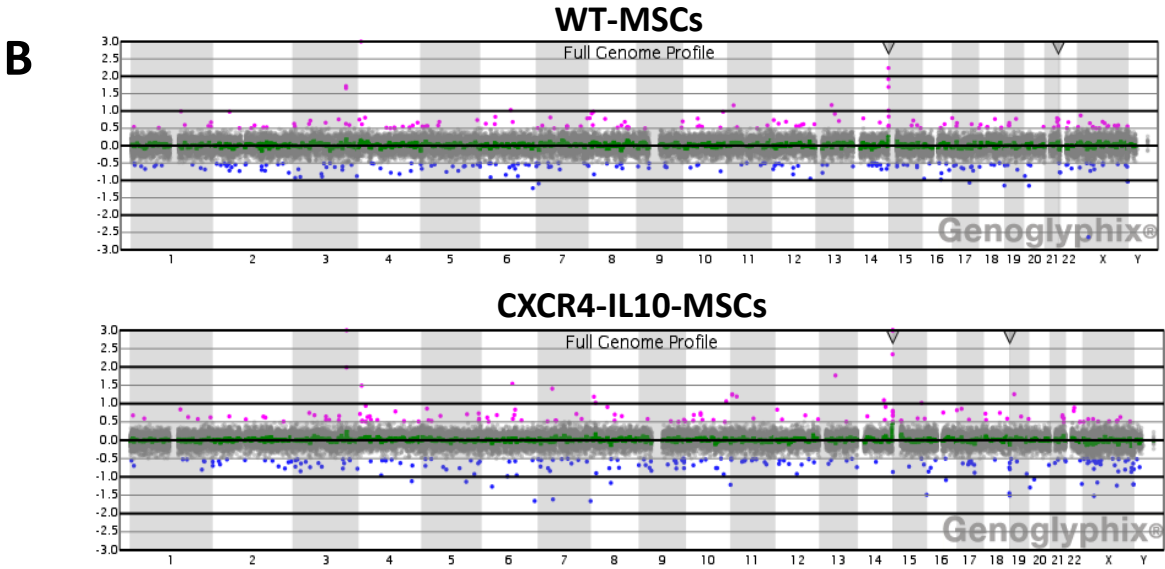

Supplement: Supplementary file 4 — Additional file 4: Figure S4. Chromosomal stability studies in WT and CXCR4-IL10-mRNA transfected Ad-MSCs. (A) Representative cytogenetic analysis in WT and CXCR4-IL10-transfected Ad-MSCs conducted 4 h after transfection. (B) Representative array CGH analysis in the same samples analyzed in panel A. [file 13287_2021_2193_MOESM4_ESM.pdf]

**Figure S5**

**A**

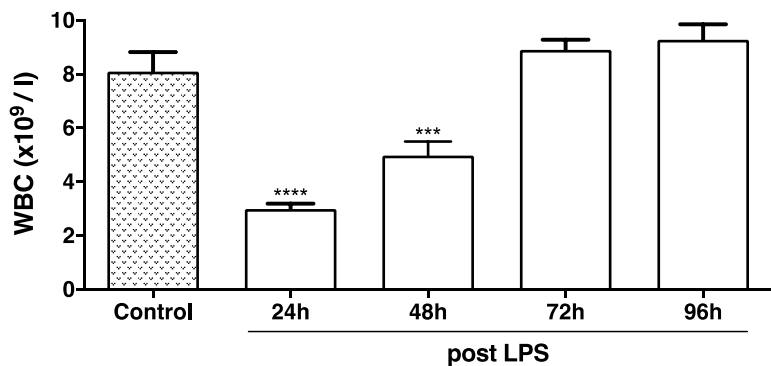

**B**

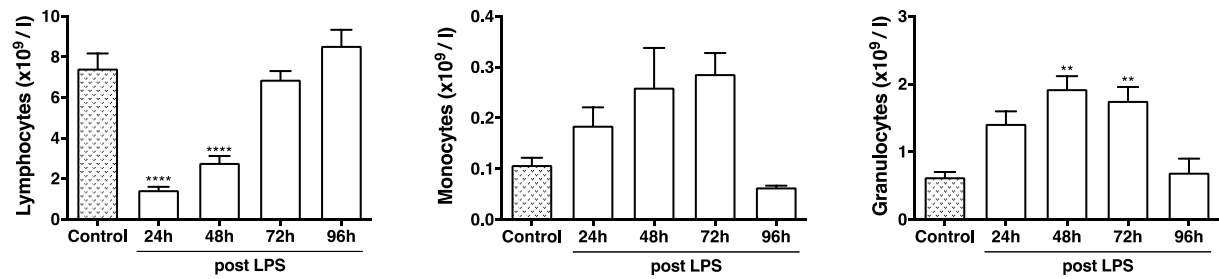

**C**

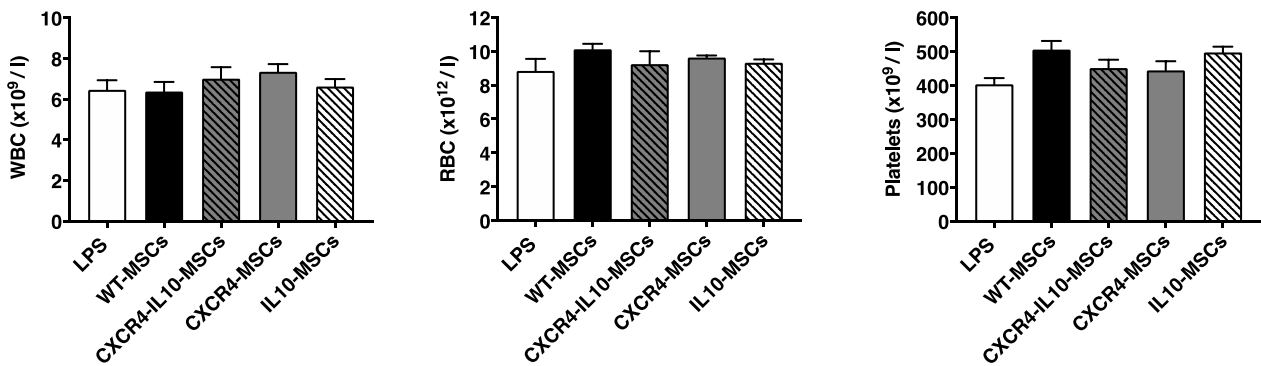

Supplement: Supplementary file 5 — Additional file 5: Figure S5. Peripheral blood cell counts in mice locally infused with LPS. (A) Evolution of circulating white blood cell (WBCs) counts in mice injected with PBS (control group) or LPS. (B) Evolution of circulating lymphocytes, monocytes and granulocytes after LPS injection compared to control mice. (C) Counts of circulating white blood cells (WBCs), red blood cells (RBCs) and platelets 72 h after LPS injection compared to levels obtained in the LPS group not receiving MSCs. Data represent the mean ± SEM of at least n = 6 mice/group. Statistical differences between LPS-injected and control mice were calculated with Tukey’s multiple comparisons test. *p < 0.05, **p < 0.01, ***p < 0.001, versus control group without LPS injection (A and B) or LPS group without MSCs (C). [file 13287_2021_2193_MOESM5_ESM.pdf]

Figure S6

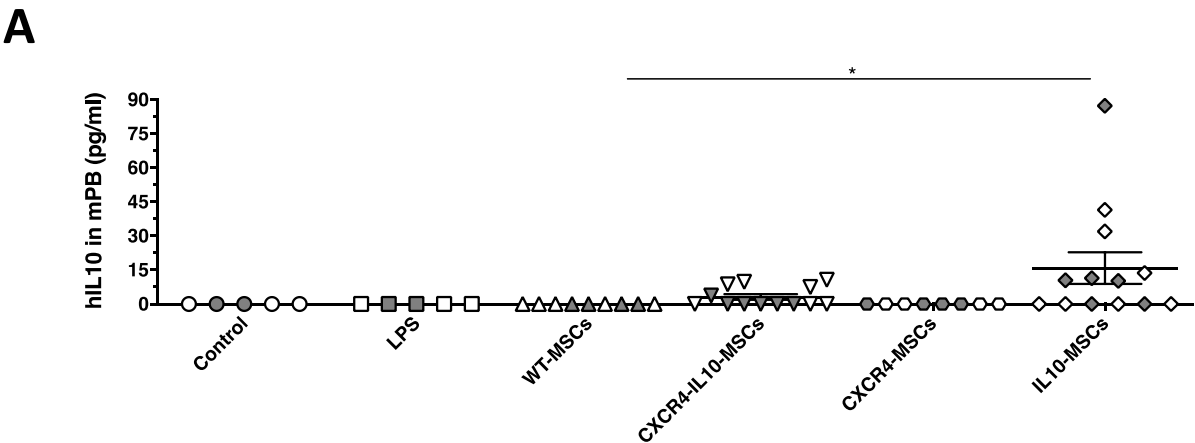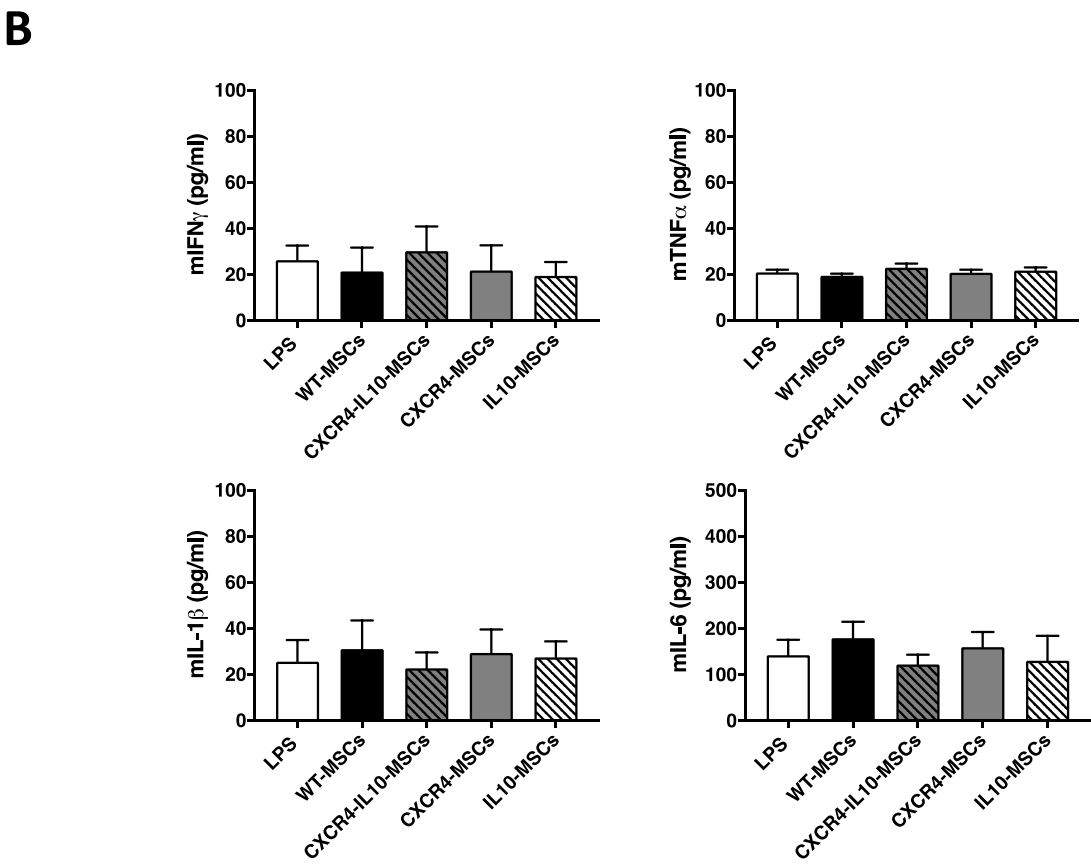

Supplement: Supplementary file 6 — Additional file 6: Figure S6. Analysis of circulating human IL10 levels in the serum of mice treated with LPS and WT or mRNA-transfected Ad-MSCs. (A) ELISA analysis of circulating human IL10 levels in the serum of mice treated with LPS and WT and mRNA-transfected MSCs. Grey and white symbols represent, respectively, analyses performed 24 or 48h after MSC infusion. (B) Levels of circulating murine IFNγ, TNFα, IL1β and IL6 in the serum of mice treated with LPS and WT and mRNA-transfected MSCs. Differences between mice receiving only LPS and mice treated with LPS plus the different Ad-MSC groups were analyzed with Tukey’s multiple comparisons test. *p < 0.05. [file 13287_2021_2193_MOESM6_ESM.pdf]

Figure S7

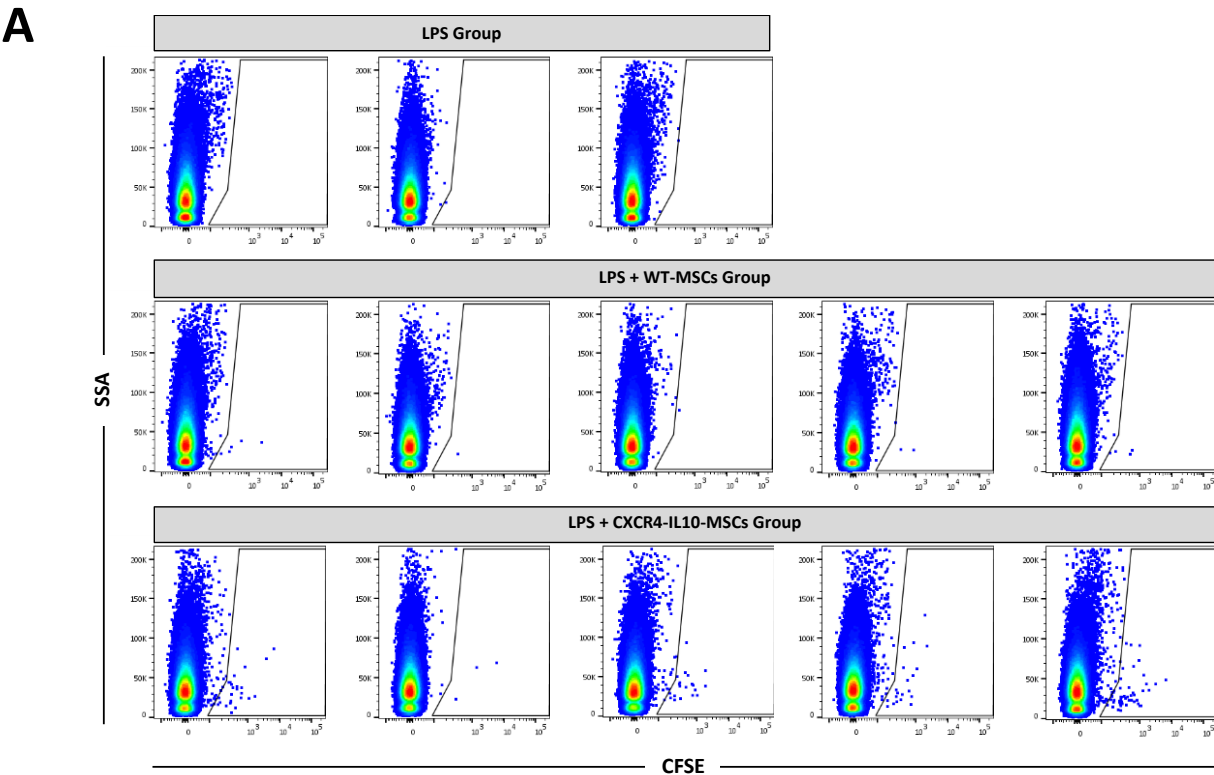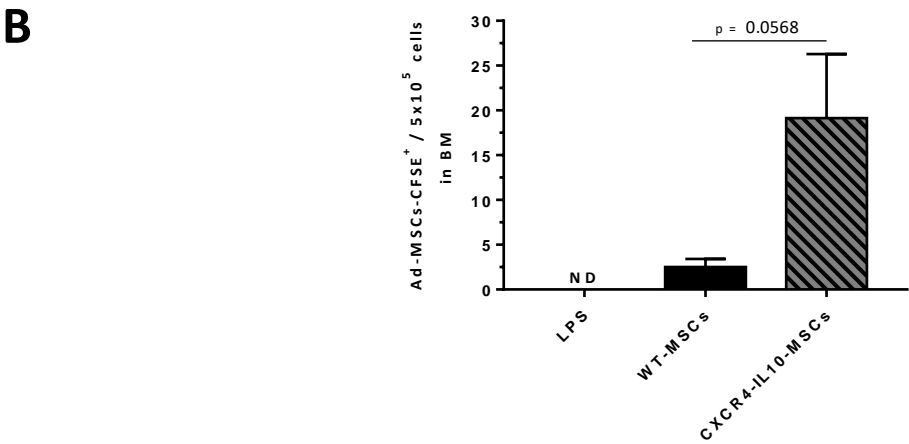

Supplement: Supplementary file 7 — Additional file 7: Figure S7. Enhanced response of Ad-MSCs co-expressing CXCR4 and IL10 to SDF1 in bone marrow of LPS-treated mice. (A) Flow cytometry analyses of CFSE-labelled Ad-MSCs in BM from mice pre-treated with LPS (local administration in one pad) and infused with WT or CXCR4-IL10-MSCs. (B) Quantification of the presence of Ad-MSCs-CFSE+ in BM. Analyses were conducted 24 hours after the infusion of Ad-MSCs. Bars represent the mean ± SEM of n = 3-5 mice/group. Unpaired t test was used to compare CFSE+ cell number in BM of mice receiving WT-MSCs with BM of those receiving CXCR4-IL10-MSCs. [file 13287_2021_2193_MOESM7_ESM.pdf]
