# Supplementary material for: Enhanced anti-inflammatory effects of mesenchymal stromal cells mediated by the transient ectopic expression of CXCR4 and IL10
Source: Stem Cell Res Ther. 2021 Feb 12;12:124. doi: 10.1186/s13287-021-02193-0 (PMC7881581; doi:10.1186/s13287-021-02193-0)
Supplement: Supplementary file 8 — Additional file 8: Table S1. Primers used in the different qPCRs. [file 13287_2021_2193_MOESM8_ESM.pdf]

Table S1

Table S1. Primers used in the different qPCRs

Name	Forward	Reverse
**CXCR4**	CCAGTTTCAGCACATCATGG	TGATACAGTAGCATGACAGGATCA
**IL10**	GCAACCCAGGTAACCCTTAAA	CATTCTTTCAGATGAAGGACCA
**ALP**	GGGTCAGCTCCACCACAA	GGCATTGGTGTTGTACGTCTT
**BGLAP**	TGAGAGCCCTCACACTCCTC	ACCTTTGCTGGACTCTGCAC
**PPARG**	GACAGGAAAGACAACAGACAAATC	GGGGTGATGTGTTTGAACTTG
**GAPDH**	GGCATGGACTGTGGTCATGA	TGCACCACCAACTGCTTAGC
