# Supplementary material for: Enhanced anti-inflammatory effects of mesenchymal stromal cells mediated by the transient ectopic expression of CXCR4 and IL10
Source: Stem Cell Res Ther. 2021 Feb 12;12:124. doi: 10.1186/s13287-021-02193-0 (PMC7881581; doi:10.1186/s13287-021-02193-0)
Supplement: Supplementary file 9 — Additional file 9: Table S2. List of up-regulated genes in mRNA-transfected Ad-MSCs compared to WT-MSCs. [file 13287_2021_2193_MOESM9_ESM.pdf]

Table S2

Table S2. List of up-regulated genes in mRNA-transfected Ad-MSCs compared to WT-MSCs.

Gene	Base Mean	FC	stat	P value	P adj
**HBB**	194261.06	4253.10	9.89	4.35E-23	6.05E-19
**CCL5**	1081.59	13.80	7.76	8.52E-15	5.93E-11
**CCL3**	386.40	30.37	7.48	7.62E-14	3.54E-10
**IL29**	66.88	37.80	5.60	2.11E-08	3.67E-05
**RTP4**	148.72	10.40	5.17	2.34E-07	2.72E-04
**IFIT1**	11611.66	11.67	4.82	1.41E-06	1.31E-03
**IFNB1**	861.10	47.74	4.85	1.23E-06	1.31E-03
**HERC5**	230.63	12.98	4.69	2.73E-06	2.24E-03
**CCL4**	25.11	Inf	4.41	1.02E-05	6.76E-03
**OAS1**	61.32	21.19	4.23	2.37E-05	1.43E-02
**TNF**	98.53	15.26	4.17	3.02E-05	1.75E-02
**IFIT2**	20591.11	13.42	4.09	4.29E-05	2.39E-02
**LRRN3**	332.99	12.53	4.07	4.76E-05	2.49E-02
**IFIH1**	870.71	12.09	3.89	1.02E-04	4.88E-02
