# Supplementary material for: Enhanced anti-inflammatory effects of mesenchymal stromal cells mediated by the transient ectopic expression of CXCR4 and IL10
Source: Stem Cell Res Ther. 2021 Feb 12;12:124. doi: 10.1186/s13287-021-02193-0 (PMC7881581; doi:10.1186/s13287-021-02193-0)
Supplement: Supplementary file 10 — Additional file 10: Table S3. List of up-regulated genes retrieving 9 KEGG pathways in mRNA-transfected Ad-MSCs compared to WT-MSCs. [file 13287_2021_2193_MOESM10_ESM.pdf]

Table S3

Table S3. List of up-regulated genes retrieving 9 KEGG pathways in mRNA-transfected Ad-MSCs compared to WT-MSCs

KEGG id	KEGG name	Genes	FDR
**hsa04620**	Toll-like receptor signaling pathway	IFNB1,CCL3,CCL4,CCL5,TNF	2.02E-04
**hsa05142**	Chagas disease (American trypanosomiasis)	IFNB1,CCL3,CCL5,TNF	3.28E-03
**hsa05160**	Hepatitis C	IFIT1,IFNB1,OAS1,TNF	6.01E-03
**hsa05164**	Influenza A	IFNB1,OAS1,CCL5,IFIH1,TNF	1.66E-03
**hsa04622**	RIG-I-like receptor signaling pathway	IFNB1,IFIH1,TNF	1.27E-02
**hsa04623**	Cytosolic DNA-sensing pathway	IFNB1,CCL4,CCL5	1.01E-02
**hsa05168**	Herpes simplex infection	IFIT1,IFNB1,OAS1,CCL5,IFIH1,TNF	2.02E-04
**hsa05323**	Rheumatoid arthritis	CCL3,CCL5,TNF	2.29E-02
**hsa04060**	Cytokine-cytokine receptor interaction	IFNB1,CCL3,CCL4,CCL5,TNF	6.01E-03
